# Narrative review on the management of moderate-severe atopic dermatitis in pediatric age of the Italian Society of Pediatric Allergology and Immunology (SIAIP), of the Italian Society of Pediatric Dermatology (SIDerP) and of the Italian Society of Pediatrics (SIP)

**DOI:** 10.1186/s13052-022-01278-7

**Published:** 2022-06-14

**Authors:** Elena Galli, Anna Belloni Fortina, Giampaolo Ricci, Nunzia Maiello, Iria Neri, Ermanno Baldo, Irene Berti, Domenico Bonamonte, Lucetta Capra, Elena Carboni, Rossella Carello, Francesca Caroppo, Giovanni Cavagni, Iolanda Chinellato, Francesca Cipriani, Pasquale Comberiati, Andrea Diociaiuti, Vito Di Lernia, Marzia Duse, Cesare Filippeschi, Arianna Giannetti, Mattia Giovannini, Amelia Licari, Gian Luigi Marseglia, Manuela Pace, Annalisa Patrizi, Giovanni Battista Pajno, Diego Peroni, Alberto Villani, Lawrence Eichenfield

**Affiliations:** 1 Pediatric Allergology Unit, Department of Pediatric Medicine, S.Pietro Hospital Fatebenefratelli, Roma, Italy; 2 Pediatric Dermatology Unit, Department of Medicine, University of Padua, Padova, Italy; 3 Department of Medical and Surgical Sciences (DIMEC), University of Bologna, Bologna, Italy; 4 Department of Woman, Child and Specialized Surgery, University of Campania “Luigi Vanvitelli”, Naples, Italy; 5 Dermatology Unit, IRCCS of Azienda Ospedaliero-Universitaria Policlinico Sant’Orsola Hospital, Bologna, Italy; 6 Giovan Battista Mattei” Research Institute, Stenico, Italy; 7 Institute for Maternal and Child Health, IRCCS Burlo Garofolo, Trieste, Italy; 8 Department of Biomedical Science and Human Oncology, Section of Dermatology, University of Bari, Bari, Italy; 9 Pediatrician, Ferrara, Italy; 10 Unit of Paediatrics, Maggiore Hospital, ASST-Cremona, Cremona, Italy; 11 Allergology Service European Diagnostic DRP Centre Parma, Parma, Italy; 12 Section of Pediatrics, S. Pio Hospital, Castellaneta, Italy; 13 Primary Care Pediatrics, AUSL Modena, Nonantola, Italy; 14 Department of Clinical and Experimental Medicine, Section of Pediatrics, University of Pisa, Pisa, Italy; 15 Dermatology Unit and Genodermatosis Unit, Genetics and Rare Diseases Research Division, Bambino Gesù Children’s Hospital, IRCCS, Rome, Italy; 16 Dermatology Unit, Arcispedale Santa Maria Nuova, Azienda USL-IRCCS di Reggio Emilia, Reggio Emilia, Italy; 17 Pediatrics, Sapienza University, Rome, Italy; 18 Dermatology Unit, Department of Pediatrics, Meyer Children’s University Hospital, Florence, Italy; 19 Pediatric Unit, IRCCS Azienda Ospedaliero-Universitaria di Bologna, Via Massarenti, 11, 40138 Bologna, Italy; 20 Allergy Unit, Department of Pediatrics, Meyer Children’s University Hospital, Florence, Italy; 21 Department of Clinical-Surgical, Diagnostic and Pediatric Sciences, Pediatric Clinic, Fondazione IRCCS Policlinico San Matteo, Pavia, Italy, University of Pavia, Pavia, Italy; 22 Clinica Pediatrica Università di Pavia - Fondazione IRCCS Policlinico San Matteo, Pavia, Italy; 23 Department of Pediatrics, S. Maria del Carmine Hospital, Rovereto, Italy; 24 Dermatology, Department of Specialistic, Diagnostic and Experimental Medicine (DIMES), Alma MaterStudiorum University of Bologna, Bologna, Italy; 25 Pediatric Unit- Policlinico Hospital, University of Messina, Messina, Italy; 26 Emergency and General Pediatrics Department Bambino Gesù Children Hospital - IRCCS, Rome, Italy; 27 Department of Dermatology, University of California, San Diego and Rady Children’s Hospital, San Diego, USA

**Keywords:** Atopic Dermatitis, Childhood, Position Paper, Management, Topical Therapies, New Drugs

## Abstract

Currently, there are a few detailed guidelines on the overall management of children and adolescents with moderate-severe atopic dermatitis. AD ​​is a complex disease presenting with different clinical phenotypes, which require an individualized and multidisciplinary approach. Therefore, appropriate interaction between primary care pediatricians, pediatric allergists, and pediatric dermatologists is crucial to finding the best management strategy. In this manuscript, members of the Italian Society of Pediatric Allergology and Immunology (SIAIP), the Italian Society of Pediatric Dermatology (SIDerP), and the Italian Society of Pediatrics (SIP) with expertise in the management of moderate-severe atopic dermatitis have reviewed the latest scientific evidence in the field. This narrative review aims to define a pathway to appropriately managing children and adolescents with moderate-severe atopic dermatitis.

## Introduction

Atopic dermatitis (AD) represents the most frequent chronic inflammatory skin disease in pediatric age with an estimated between 16 and 20%. The health and social costs of the disease are relevant not only for the complexity of pharmacological treatments, but also for the very frequent complications, for the strong impact on the psychological balance of families and on the times of assistance required, on the quality of life of the sick and family members, on sleep disorders and on attention disorders and irritability that follow [1]. Erythematous lesions, papules and vesicles, crusty scratching lesions for severe itching, lichenification and xerosis are the clinical features whose severity is measured through various objective scales such as the SCORAD index [2] (Severity ScoRing of Atopic Dermatitis) or EASI [3] (Eczema Area and severity index)

AD displayed a growing body of new comorbidities also in dermatology and oral diseases (4-%). An observational study conducted by Perugia et al [4, 5], found a higher prevalence of atopic dermatitis in pediatric dentistry patients compared to the general population suggesting that dental diseases could be involved in the pathogenesis of AD. Furthermore that actually Italia guidelinestend [6, 7] to be more anymore inclusive for pediatrics, however a dedicated document is mandatory to highlight knowledge gaps.

Experts of the Italian Society of Pediatric Allergology and Immunology (SIAIP), the Italian Society of Pediatric Dermatology (SIDerP), and the Italian Society of Pediatrics (SIP) have updated the management of AD in the light of the most recent pathogenetic and therapeutic findings

## Methods

A joint Task Force of experts of the SIAIP, SIDerP, and SIP defined the topics to address in the review (appendix 1)

A literature search was performed in September 2021 across MEDLINE/PUBMED to identify studies investigating the management of moderate-to-severe AD in the pediatric age. We included randomized controlled trials (RCTs), observational (cross-sectional and cohort), case-control studies, and systematic review and meta-analyses, which [8] were written in English, [9] included patients 0-18 years of age with moderate-severe AD, either isolated or associated with other atopic comorbidities, [10] reported systemic monotherapy or systemic therapy with topical anti-inflammatory therapy (combination therapy) or topical anti-inflammatory therapy alone, and [11] reported efficacy and/or safety. The search criteria were atopic dermatitis* OR atopic eczema* OR eczema* AND severe* OR moderate-severe* OR therapy* OR treatment* OR azathioprine* OR ciclosporin* OR methotrexate* OR mycophenolate* OR interferon-gamma* OR upadacitinib* OR baricitinib* OR dupilumab* OR dupixent* OR abrocitinib* or tralokinumab* OR nemolizumab* OR lebrikizumab* OR biologic* OR biological* OR topical* OR corticosteroid* OR glucocorticoid* OR calcineurin inhibitor* OR immunomodulator* OR immunosuppressant* OR tacrolimus* OR pimecrolimus* OR wet-wrap* OR Janus Kinase inhibitor* OR antibodies* OR monoclonal* OR antimicrobial* OR antibiotic* OR antiviral* OR antihistamine* OR emollient* OR moisturizer* OR phototherapy* OR immunotherapy* OR education* OR intervention*. Titles and abstracts of citations identified from searches and content of relevant full texts were evaluated. Studies that did not specifically measure the severity of AD, using either the Eczema Area and Severity Index (EASI), or Investigator’s Global Assessment (IGA), or the SCORing Atopic Dermatitis (SCORAD), were excluded. The special interest was in studies published within the last 36 months. The screening was conducted by 2 investigators (one pediatric allergist and one pediatric dermatologist), with a third investigator (EG) resolving any disagreements.

## Conclusions

In recent years, important insights into the pathogenesis of AD have been observed; this aspect was evidently reflected in drug therapy; in particular the moderate-severe forms are those in which we have had the greatest therapeutic innovations. The purpose of this narrative review was to update knowledge in the management of AD with a particular reflection on those situations where biological drugs may be involved. Having combined the knowledge of pediatricians, allergists and dermatologists should allow us to have a valid document for all those who deal with AD in pediatric age.

## Data Availability

Not applicable.

## Appendix 1

### Topics addressed in the review

1. Topical Corticosteroids

2. Topical calcineurin inhibitors

3. Antibiotic, antiseptic, antiviral, and antifungal therapy

4. New topical therapies: Topical phosphodiesterase 4 inhibitors and Jak kinase inhibitors

5. Wet Wrap dressing

6. Special fabrics

7. Systemic Corticosteroids

8. Cyclosporin A

9. Methotrexate

10. Azathioprine

11. Mycophenolate mofetil

12. Antihistamines

13. Probiotics

14. Phototherapy

15. Dupilumab

16. New Drugs

17. Allergen Specific Immunotherapy

18. Thermal Therapy

19. Therapeutic education

### Topical corticosteroids

Topical corticosteroids (TCSs) are currently recommended as a first-line anti-inflammatory therapy for AD [3, 8–10]. The efficacy and availability of molecules with different potency and in different formulations have contributed to the widespread use of TCSs. TCSs exert an anti-inflammatory, antiproliferative, immunosuppressive, and vasoconstrictive action [11, 12]. TCSs have direct regulatory effects at the cellular level by binding to their receptor to form a corticosteroid-receptor complex capable of translocating within the nucleus and stimulating or inhibiting the synthesis of proteins. In addition, they can indirectly regulate transcription by blocking the effect of other transcription factors [12]. TCSs have been shown to inhibit the transcription of various pro-inflammatory cytokines involved in skin diseases (e.g. interleukin (IL)-1, IL-2, IL-6, TNF-α, and interferon-gamma (INF-γ) and stimulate the expression of genes that encode anti-inflammatory cytokines such as TGF-beta and IL-10. Through this immunomodulatory activity, TCSs probably play an effective role in balancing the ratio of Th1 to Th2 lymphocytes at the level of skin lesions [12]. Other anti-inflammatory effects include acceleration of apoptosis of eosinophils and T lymphocytes, suppression of the function of endothelial cells and lymphocytes, inhibition of dermal edema and dilation of capillaries, and reduction of vascular permeability. Finally, TCS showed anti-proliferative effects on several cells, including T lymphocytes [11].

In choosing the TCS to use in a child with AD, several factors must be taken into consideration such as the potency of the drug, the delivery vehicle, the age of the patient, the site and the body surface to be treated. Based on their potency, TCSs are classified in various ways. In Europe, they are divided into four groups, where group 1 contains the low potent TCSs, while group 4 contains the very potent TCSs (Table 1).

**Table 1:** Potency classification of topical corticosteroids (from patrizi et al, [13])

**Group/Potency**	**Active ingredient**	**Vehicle**	**Concentration (%)**
**Group I - Mild**	Hydrocortisone	cream	0.5
	Hydrocortisone acetate	cream	0.5
**Group II - Moderate**	Aclomethasone dipropionate^a^		
	Clobethasone butyrate	cream	0.5
	Dexamethasone sodium phosphate	ointment	0.2
	Dexamethasone valerate	cream	0.1
	Desonide^a^		
	Fluocortinbutylester^a^		
	Hydrocortisone butyrate	cream, cream hydrophilic, emulsion, cutaneous solution, ointment	0.1
**Group III - Potent**	Beclomethasone dipropionate	cream	0.025
	Betamethasone benzonate	cream	0.025
		cream, skin emulsion, gel	0.1
	Beclomethasone dipropionate	cutaneous solution	0.05
		cream, skin emulsion, ointment	0.1
	Betamethasone dipropionate	cream, cutaneous solution, ointment	0.05
	Budesonide	cream, oinment	0.025
	Deoxymethasone	emulsion	0.25
	Diflucortolone valerate	cream, cream hydrophobic, cutaneous solution, ointment	0.1
		cream hydrophobic, ointment	0.3
	Diflucortone valerianate^a^		
	Fluocinolone acetonide	cream	0.025
		cutaneous solution	0.01
	Fluocinonide	cream, gel, cutaneous solution	0.05
	Fluocortolone pivalate / caproate	cream hydrophobic	0.25
		cream	0.25
	Fluticasone propionate	oinment	0.005
		cream	0.05
	Methylprednisolone aceponate	cream, cream hydrophobic, emulsion, cutaneous solution ointment	0.1
	Mometasone furoate	cream, cutaneous solution, ointment	0.1
	Prednicarbato	cream	0,25
**Group IV – Very potent**	Alcinonide	cream	0.1
	Clobetasol propionate	oinment	0.05

^a^molecules not available in Italy

In the US, the classification includes seven groups, where group 1 contains the most potent TCSs, while group 7 contains the least potent TCSs [8]. In Japan, the classification includes five groups and the potency of TCSs decreases as the number of the group increases [9]. The potency of the TCS to be used should be chosen based on the degree of severity of AD, favoring moderately potent and low potent TCSs for mild cases of eczema, and reserving potent TCSs for severe and moderate cases of AD [9]. Very potent TCSs (e.g. clobetasol propionate) should be avoided in children as their use carries a significantly higher risk of adverse effects, both local and systemic, than TCSs from other groups [14]. The early use of a TCS of adequate potency, in sufficient quantity, and at the beginning of the flare-up of AD, increases the likelihood of controlling inflammation, restoring the skin barrier and reducing the subsequent need for TCSs [15, 16].

When prescribing TCSs, it is also important to choose the adequate delivery vehicle, which must be selected based on the characteristics of the lesions and the skin site, and the patient's preferences and tolerability, in order to ensure adherence to therapy.

The delivery vehicle, in addition to being the carrier of the pharmacological active principle, plays an important role in determining its bioavailability. To be active, a vehicle must be able to maintain the solubility and stability of the pharmacological active principle, release the drug and distribute it adequately in the skin, allow penetration through the skin barrier, guarantee the pharmacological effect of the active ingredient and limit its systemic absorption [17]. Depending on the type of vehicle used, the effects of a given active ingredient can vary in terms of potency and clinical efficacy [17]. Creams, which are often enriched with humectant molecules to enhance the moisturizing effect, are indicated for the treatment of acute or subacute lesions; ointments are instead indicated to treat areas of chronic lesions (e.g. lichenified and particularly xerotic lesions) and with a thick corneal layer (e.g. palm / plantar regions), given the high lipid content and high occlusive properties [17].

The Fingertip Unit (FTU) is the unit of measurement that has been in use for years to determine the right amount of TCS to be applied [18]. This amount corresponds to approximately 0.5 g and is sufficient to cover an area of skin equal to two palms of an adult's hand. Table 2 shows the various FTUs needed to treat the different skin surfaces according to the age of the child.

**Table 2:** Adequate doses of topical corticosteroids to be applied in finger tip unit (ftu) (modified from katoh et al, [2])

**1 FTU = 0.5 g**					
**Child**	**Face and neck**	**Upper limb**	**Lower limb**	**Trunk**	**Back**
3-6 months	1	1	1,5	1	1,5
1-2 years	1,5	1,5	2	2	3
3-5 years	1,5	2	3	3	3,5
6-10 years	2	2,5	4,5	3,5	5
**Adult**	**Face and neck**	**Upper limb**	**Lower limb**	**Trunk**	**Back**
	2,5	3+1	6 + 2	7	7

The use of this method allows the healthcare professional to have a more precise idea of the dose of TCS prescribed and the parent to overcome the increasingly widespread concern about the risk of an overdose of corticosteroids. This helps to counteract so-called corticophobia, promote therapeutic adherence and avoid under-dosage of TCSs.

The appropriate use of TCSs, even in the long term, may only rarely cause the adverse effects reported in the past (e.g. skin atrophy, striae rubre, telangiectasias). A cross-sectional observational study involving 70 children with a mean age of 3.2 years, did not document any degree of skin atrophy following an average use of about 11 months of a combination of potent TCS (betamethasone dipropionate 0.05% ointment, methylprednisolone aceponate 0.1% ointment or mometasone furoate 0.01%), moderate potency (betamethasone valerate 0.02% ointment) and low potency (hydrocortisone acetate 1% ointment). In this study, the mean monthly dose of TCS was 79 g, 128 g, and 34 g for potent, moderate potency, and low potency TCS, respectively [19]. Even in the short term, the use of TCS appears safe in terms of local adverse effects. In another study involving 174 children with AD treated with a 3-day course of a potent TCS (betamethasone valerate 0.1%), no skin thinning was documented on skin ultrasound [20]. Among the various molecules, mometasone furoate, methylprednisolone aceponate, hydrocortisone aceponate and betamethasone valerate have little or no atrophying effects [20, 21].

A few studies have evaluated the systemic effects of TCSs, such as suppression of the hypothalamic-pituitary-adrenal axis. In a meta-analysis that analyzed a total of 12 trials and 522 children, evidence of biochemical suppression of the hypothalamic-pituitary-adrenal axis, assessed at 2-4 weeks, was documented in 2%, 3.1% and 6.6% of children who used low, medium and high potency TCSs, respectively [22]. However, the normal endocrine function was restored 1-10 weeks after discontinuation of therapy. Another study showed that using a potent TCS (fluticasone dipropionate 0.05% cream) twice a day for 3-4 weeks over a large body surface area (on average 64%) in two groups of patients (3 months-3 years and 3-6 years of age) and with a mean dose over the study period of 96.7 g in the first group and 209.1 g in the second group, had a low risk of suppression of the hypothalamic-pituitary-adrenal axis [23]. In particular, of the 43 children studied, only 2 (4.7%) showed a suppression of the axis, also in this case asymptomatic and reversible.

On the other hand, currently available data on the effects of long-term therapy with TCS are scarce. The Petite Study [13], is one of the few studies in which a large follow-up period was used. In this randomized trial, the authors compared the safety of using topical pimecrolimus vs TCS on 2439 children divided into two groups (1210 were treated with pimecrolimus and 1229 with TCS). Participants were followed up to 5-6 years of life with a particular focus on the effect on the trend of stature growth and the immune system. The TCSs used in this study were low potency (e.g. hydrocortisone 1% cream/ointment) and/or medium potency (e.g. hydrocortisone butyrate 0.1% cream/ointment) TCSs, with exposure to TCS therapy ranging from 77- 396 days (although the average dose taken per time interval is not specified). The effect on stature growth and the development and maturation of the immune system was the same for the two groups of patients. The analysis of the available data suggests that TCS are sufficiently safe drugs in terms of systemic adverse effects in long-term therapy, but to date, it is not possible to establish a "safe" recommendable dose (for example as a monthly cumulative dose), due to the scarcity and poor homogeneity of data. Likewise, it is difficult to recommend the "best" TCS. However, the newer generation of TCSs (i.e. the non-halogenated double esters) has a better risk/benefit ratio, as it can balance a powerful anti-inflammatory effect with reduced systemic toxicity and a low atrophying potential [24]. Furthermore, it is not clear that two administrations per day are more effective than a single application 10). Therefore, the number of daily administrations may be decided according to the severity of the lesions: twice a day in the severe acute phase, and once a day in the mild forms or remission [9, 10].

Once remission of skin lesions is achieved, TCSs should be gradually decreased or discontinued. In this regard, a useful therapeutic strategy is termed proactive therapy. It consists of the application of TCSs twice a week (eg weekend therapy) in the skin areas of most frequent recurrence, despite the absence of active lesions, in order to maintain remission [8, 9, 14].

This therapeutic strategy is particularly effective in patients who have frequent flare-ups, which are reduced [8, 25]. The choice of potency, vehicle and amount of TCS should also be based on the skin region of application. The sites with the greatest absorption of TCSs, such as the eyelids, genitals, face and skin folds [17], require careful monitoring of possible side effects. Prolonged use of TCSs on these skin areas should be avoided, especially if they are of moderate-high potency [9]. Also, adverse intra-ocular effects (eg glaucoma and cataracts) which can occur with the use of TCS in the periorbital region, are rare and may be limited by the use of potent or moderately potent TCSs only for short periods. If maintenance therapy is required in this skin region, the use of low-power TCS or topical calcineurin inhibitors (TCIs) is recommended.

Regarding the risk of osteopenia/osteoporosis, the available data suggest that, in children treated with TCSs, the expected prevalence of low bone mineral density is the same as in the general population [26].

Despite the robust safety profile of TCSs, corticophobia - that is excessive worry, fear and reluctance about the use of TCSs - is increasing among caregivers. It is estimated that corticophobia affects up to 60-73% of patients or parents of children with AD [27] and that it represents one of the most important causes of non-adherence to therapy, partly due to insufficient explanations by healthcare professionals [28]. Given the magnitude of the problem, it is important to promote the practice of a standardized assessment of corticophobia and strengthen the therapeutic education strategies of the patient and families. A tool that can be used in the evaluation of corticophobia is the Topical Corticosteroid Phobia (TOPICOP) score, conceived by Aubert-Wastiaux in 2013 [29] which consists of 12 items organized in the form of a questionnaire aimed at evaluating two different areas: fears and beliefs about TCSs. A standardized evaluation of corticophobia, ideally to be performed at the first visit of a new patient with AD, can be an important tool to improve adherence to future therapy with TCSs [28].

### Topical calcineurin inhibitors

In the years 2000-2001, two TCIs, tacrolimus (tTAC) and pimecrolimus (tPIM), were approved for the treatment of AD in patients ≥ 2 years of age. TCIs are non-steroidal anti-inflammatory agents that selectively inhibit the phosphatase activity of calcineurin, which leads to reduced transcription of various pro-inflammatory cytokines involved in the development and maintenance of inflammation in AD, such as IL-2, IL-3, IL-4, IL-5, INF-γ, and granulocyte-macrophage colony-stimulating factor (GM-CSF) (Table 3) [30–32].

**Table 3:** Main effects of topical calcineurin inhibitors compared to topical corticosteroids

**Parameter**	**Topical Pimecrolimus (810 Da)**	**Topical Tacrolimus (822 Da)**	**Topical Corticosteroids (<500 Da)**
Activity on cells	T Lymphocytes, Mast cells,	T Lymphocytes, Mast cells, Eosinophils, Basophils, Langherans cells,	T Lymphocytes, Mast cells, Eosinophils, Basophils, Langherans cells, Keratinocytes, Endothelial cells, Fibroblasts
Cytokines	IL-2, IL-3, IL-4, IL-5,IL-13,IL-33, TNF-α, INF-γ, GM-CSF	IL-2, IL-3, IL-4, IL-5,IL-13,IL-31,IL-33, TNF-α, INF-γ, GM-CSF	IL-1,IL-2, IL-3, IL-4, IL-5,IL-6, IL-13,IL-31,TNF-α, INF-γ, GM-CSF,TSLP
Inhibition function and Apoptosis of Langherans cells	-	+	++
Absorption through the skin	+	++	+++
Atrophogenic activity	-	-	+++

TCIs have also been shown to down-regulate the expression of the high-affinity receptor for immunoglobulin E class (FcεRI) on Langerhans cells, suppress the T cell proliferation induced by *S. aureus* toxins and the expression of IL-33 mRNA, triggered by enterotoxin B, and correct the altered balance of Toll-like receptors [31, 33]. Some data suggest that tTAC can also directly act on sensory nerves and inhibit their activation by inducing sensory desensitization and suppression of the release of substance P [34]. A 10-year follow-up study reports that tTAC significantly reduces the body surface area affected by AD and serum IgE levels, and in those individuals with active asthma and rhinitis, tTAC can also reduce respiratory symptoms and bronchial hyper-reactivity [35].

Compared to tTAC, tPIM shows lower skin penetration, higher affinity to epithelial structures, lower affinity to lymphoid structures and lower immunosuppressive effects [36]. Regarding pharmacokinetics, the cutaneous absorption of TCIs is minimal due to their large molecular size, respectively 822 Dalton (Da) for tTAC and 810 Da for tPIM. The transepidermal penetration of TCIs is 70-100 times lower than that of topical corticosteroids (TCSs), with tPIM in cream showing approximately five times lower transepidermal flow than tTAC in ointment [37].Conversely, CSTs have a molecular weight <500 Da and are therefore more absorbed even by healthy skin [36, 37]. The maximum absorption of TCIs is observed in the initial stages of treatment, when the inflammation is greater, while, when the inflammation is reduced and the skin barrier begins to improve, their penetration further decreases [36, 37]. There is conflicting evidence regarding TCIs effects on the skin barrier, with recent studies indicating the superiority of tTAC over CSTs in restoring skin barrier integrity [38, 39].

#### Effectiveness

TCIs are safe and effective if used in sufficient dosage and with correct application, as amply demonstrated in the last twenty years by numerous studies and meta-analyses, both in the pediatric and adult populations [40–43].

In a recent systematic review of the efficacy and safety of TCSs compared to TCIs, Siegfried et al. [44] demonstrated that the data supporting the long-term use of TCIs are particularly robust, while those supporting the use of TCSs are limited to low-medium power products. In a 2018 review article, which included the combined results of 19 studies on the use of TCIs in patients with AD, the two formulations of tTAC (0.03 and 0.1%) were more effective than low-power TCSs, with an efficacy at least similar to that of medium power TCSs [8, 45].

ETFAD recently recommended the use of TCIs as the first choice in sensitive areas of the body, with a preference for tPIM in mild AD and tTAC in moderate-to-severe AD and long-term treatments, as well as their use off- label in children under 2 years of age [45]. A very recent European Expert Panel concludes that since the treatment of AD should be started from a very early age and that tPIM is a safe and effective "steroid-sparing" treatment option in both the short and long term, this drug should no longer be denied to children aged 3 months or olde [46].

TCIs significantly relieve itching, which is reduced already after the first days of treatment, as evidenced in a meta-analysis [47]. TCIs can be used as a maintenance treatment to minimize the use of TCSs in patients whose disease has stabilized. The application of tTAC 2-3 times a week for up to 1 year increases the number of days without acute AD lesions and lengthens the time of AD exacerbation [48, 49]. Recent data also suggest that TCIs may have a positive impact on the altered skin microbiome, as they reduce S. aureus colonization and increase microbial diversity in lesional areas of the skin [50].

TCIs have been described to cause neither cataracts nor glaucoma, which would make them particularly useful in treating eye allergies [51].

The indications and contraindications of TCIs are shown in Tables 4 and 5.

**Table 4:** Indications and contraindications for topical tacrolimus (ttac) 0.03% and 0.1% ointment

**Indications**
• Moderate to severe dermatitis in sensitive body sites (first choice)
• Moderate to severe atopic dermatitis in which (one of the following applies): a) there is no response to first-line therapy with TCSs; b) there are contraindications to treatment with TCSs; c) undesirable effects induced by the use of TCSs may occur, such as skin atrophy or telangiectasia; d) Long-term maintenance therapy is required. *Tacrolimus is approved for maintenance therapy to prevent relapses of AD and prolong intervals without flare-ups in patients experiencing a high frequency of relapses. In all these patients, sunscreen should be encouraged to reduce a hypothetical risk of photocarcinogenesis.*
**Contraindications**
**Absolute**
Hypersensitivity to tTAC or other components of the ointment
**Relative**
a) children aged <2 years (0.03% concentration is indicated for age 2- <16 years; 0.1% concentration is indicated for ≥ 16 years). The use in this age group is off-label, but with various studies supporting the safety of use at age <2 years.
b) active skin infections (viral and/or bacterial) in place at the application site
c) eroded or ulcerated surfaces at the application site (if they were present in multiple forms, the application of this ointment should be started after the improvement of the lesions obtained with TCSs).
d) immunocompromised patients primitively, secondarily, or taking immunosuppressive drugs and/or with neoplasms
e) any lymphadenopathies present at the time of starting therapy should be evaluated and kept under observation
f) should not be used under occlusive dressing
g) the combination with phototherapy is not recommended

tTCA should not be applied to the skin within two hours of applying an emollient cream

**Table 5:** Indications and contraindications for topical pimecrolimus (tpim) 1% cream

**Indications**
• Mild to moderate dermatitis in sensitive body sites (first choice)
• Moderate to severe atopic dermatitis in which (one of the following applies): a) there is no response to first-line therapy with TCSs; b) there are contraindications to treatment with TCSs; c) undesirable effects induced by the use of TCSs may occur, such as skin atrophy or telangiectasia; d) Long-term maintenance therapy is required. *Pimecrolimus is approved for maintenance therapy to prevent re-ignition and prolong intervals without flare-ups in patients experiencing high relapse rates. In all these subjects, sunscreen should be encouraged to reduce a hypothetical risk of photocarcinogenesis*
**Contraindications**
**Absolute**
Hypersensitivity to tPIM or other components of the cream
**Relative**
a) children aged <2 years (0.03% concentration is indicated for age 2- <16 years; 0.1% concentration is indicated for ≥ 16 years). The use in this age group is off-label, but with various studies supporting the safety of use at age <2 years.
b) active skin infections (viral and/or bacterial) in place at the application site
c) eroded or ulcerated surfaces at the application site (if they were present in multiple forms, the application of this ointment should be started after the improvement of the lesions obtained with TCSs).
d) immunocompromised patients primitively, secondarily, or taking immunosuppressive drugs and/or with neoplasms
e) any lymphadenopathies present at the time of starting therapy should be evaluated and kept under observation
f) should not be used under occlusive dressing
g) the combination with phototherapy is not recommended

tPIM should not be applied to the skin within two hours of applying an emollient cream

If no improvement occurs after 2 weeks of treatment, compliance and the possible influence of irritants should be carefully evaluated before considering alternative therapeutic options [29].

Furthermore, TCIs are an effective therapeutic option for pediatric patients with perioral atopic dermatitis, as recently shown in a study involving 132 children (mean age 4.2 years). Monitoring of TCIs blood levels is not currently recommended [52].

#### Side effects

The most reported side effects are application site reactions (e.g. burning, prickling sensation on the skin, itching, and erythema), which are more frequent during the first few days of application. Due to the release of neuropeptides, the irritative effects may be more persistent and be aggravated by intense sweating and alcohol intake in some patients. To remedy the burning sensation, one can start with the application of TCSs followed by the tTAC at 0.03% and then continue with tTAC at 0.1% if the age of the patient allows this formulation. It is advisable to keep the drug in the refrigerator at 5-7 degrees, as cooling reduces the skin vascular instability and the infiltration of inflammatory cells. There are some reports of allergic contact dermatitis or rosacea-like granulomatous reaction or melanosis of the lips with the use of TCIs [45]. Viral infections, such as *eczema herpeticum* or *eczema molluscatum*, have also been observed during treatment with TCIs.

#### Evidence for safety and tolerability

In 2005 in the USA the Food and Drug Administration (FDA) issued a "Black Box warning" relating to a theoretical risk of skin cancers and/or lymphoma related to the application of TCIs. However, it is now clear that the evidence used for the Back Box warning was insufficient to establish a causal relationship. To date, there is no scientific evidence of the association between the use of TCIs and a higher incidence of skin cancers or lymphomas in patients with AD [53].

In 2018, a multicenter cohort study evaluating a very high number of children and adults treated with TCIs vs untreated patients concluded that the use of tTAC and tPIM was associated with an increased risk of lymphoma but with only a small excess risk for individual patients. However, in this study, there are residual confounding factors, such as the severity of AD, increased monitoring of severe patients, and inverse causality that may have influenced the results [54].

Immediately after the Black Box, two important registries were created to assess the risk of malignant tumors in pediatric age, the Pediatric Eczema Elective Registry (PEER) [55] which recruited children aged 2-17 years treated with tPIM, and the APPLES (A Prospective Pediatric Longitudinal Evaluation to Assess the Long-Term Safety of Tacrolimus Ointment for the Treatment of Atopic Dermatitis) [56] in which children up to 16 years of age treated with tTAC for a period ≥ 6 weeks. Both registries, which included a very large number of patients, concluded that the incidence of cancer in these patients was no different from that predicted for age. Therefore, for both drugs, there is no support for the initial hypothesis that they increase the risk of long-term cancer in children with AD [55, 56].

A further and recent study has also highlighted a relationship between allergic diseases, use of corticosteroids and Hodgkin's lymphoma, reporting how immunosuppression was associated with a 6 times greater probability of lymphoma, with minimal change after adjustment for the use of corticosteroids [57]. Furthermore, it has recently been shown that there is no relationship between the risk of keratinocyte tumors and the use of TCIs [58]. Finally, it should be noted that pediatric studies also support the lack of systemic immunosuppression by TCIs used both for short- and long-term treatments [13].

### Antibiotic, antiseptic, antiviral, and antifungal therapy

#### Antibiotic therapy

Patients with AD are more susceptible to secondary bacterial, fungal, or viral skin infections, which may be isolated or superimposed, and at risk for systemic spread. Factors contributing to the risk of skin infections are mainly related to endogenous (skin barrier damage and non-acid pH of diseased skin) and exogenous (irritating agents, scratching, climatic-environmental factors) factors. The acidic pH of healthy skin reduces the expression of staphylococcal surface proteins, such as clumping factor B and fibronectin-binding protein, which bind to host proteins (cytokeratin 10 and fibronectin). Defects in the expression of the filaggrin gene lead to a decrease in the levels of urocanic acid and carboxylic pyrrolidone acid, with a further increase in skin pH, and to the down-regulation of innate and adaptive skin immunity, thereby facilitating colonization by the S. aureus [59]. The activation of various cytokines (IL-4, IL-13, TSLP) induces a reduction of antimicrobial peptides, such as cathelicidin LL-37, dermocidin and ß-defensins, thus favoring skin colonization by pathogenic microorganisms, such as S. aureus.

S. aureus can enhance skin inflammation, by releasing superantigenic toxins, and itching by activating cutaneous T lymphocytes, through IL-31 [60–62]. Exacerbations of AD are associated with loss of diversity in the skin microbiome, contrary to what occurs in patients on proactive therapy [45, 63, 64]. Other commensal bacteria, such as *Staphylococcus epidermidis* (*S. epidermidis*) and *Staphylococcus hominis*, are also able to modulate the development of skin T cells, inhibit inflammation and prevent skin infection by producing antimicrobial peptides (AMPs) [65]. *Streptococcuss Spp*. and *S. aureus* can coexist in 70-80% of skin cultures from patients with AD and infected lesions [66, 67].

Topical antibiotics should be used when there is clear evidence of secondary infection, selecting molecules with good efficacy against *Staphylococci* and *Streptococci*. The most frequently used topical antibiotics are fusidic acid and mupirocin. Retapamulin is also effective but is currently out of the market in Italy. In Europe, ozenoxacin has recently been approved for the treatment of nonbullous impetigo in adults and children over 6 months. It is a new non-fluorinated quinolone, with a broad spectrum of action against *Streptococci* and *Staphylococci*, even for species resistant to mupirocin and methicillin. Therefore, it is believed that this antibiotic may represent a valid alternative to treat impetiginized lesions of AD [68].

Topical antibiotics can induce allergic contact dermatitis. In this regard, fusidic acid has shown a very low ability to induce sensitization, despite being used for several years [69, 70]. Topical antibiotics can also cause irritant contact dermatitis, generally caused by excipients and/or preservatives contained in the topical preparation (eg lanolin, cetyl alcohol, stearyl alcohol). The duration of treatment should therefore be limited to the resolution of the impetigo to prevent any risk of sensitization and/or development of drug resistance.

Fusidic acid and mupirocin should ideally be applied 2-3 times a day for 7-10 days. The treatment should not be extended beyond 10 days; furthermore, mupirocin should not be used in children under the age of 1 year, due to the lack of studies in this age group [71]. The recurrence of infections in patients with AD is frequently associated with nasal colonization by *S. aureus*. When the nasal swab is positive, nasal decolonization with mupirocin (after performing the antibiogram) has been shown to be effective, with 2 applications per day in both nostrils for 5 days per month, for 3 months, to be performed in all family members (who are often asymptomatic carriers) [72]. Pets can harbor *S. aureus*, including MRSA, and it is advisable to seek advice from an experienced veterinarian for the evaluation and management of the animal [73] erall improvement in the signs and/or symptoms of AD compared to using the topical steroid alone [74].

Systemic antibiotic treatment should be combined with topical therapy only in case of bacterial superinfection extended to more than 2% of the skin surface, poor response to topical treatment, or tendency to frequent relapses. Based on current resistance spectra, cephalexin, or another first-generation cephalosporin, may be recommended for 7-10 days [74–76]. In cases of AD with widespread impetiginization (*Eczema staphylococcatum*) [77] or relapsing, the combination amoxicillin-clavulanate or a fluoroquinolone can be administered [78]

Skin swab cultures should be reserved for patients suspected of having an *Methicillin-resistant Staphylococcus aureus* (MRSA) infection. In case of a high rate of MRSA infection in the community (> 10%), oral clindamycin or trimethoprim-sulfamethoxazole can be used for 7-10 days (at least until complete resolution). Overall, antibiotics may be less effective due to the development of resistant strains, recolonization, and their negative impact on commensal microbes [79].

#### Antiseptic therapy

Proper cleansing is the first step to prevent skin superinfections. If there is no response to topical therapy with TCSs or TCIs and/or in the presence of an evident skin infection, the use of topical antiseptics may be considered.

Diluted bleach baths are the most frequently used antiseptic remedy. These baths can be used daily in the most serious patients or the form of hand baths or foot baths in case of more recalcitrant localized lesions. Intermittent use of 0.005% sodium hypochlorite (NaOCl) (100 ml of 5% bleach added to 100 L of bathtub water) showed a significant decrease in AD severity.

A recent open-label prospective study conducted on 50 patients (aged 6 months-17 years) suffering from moderate-severe AD with documented colonization by S. aureus, showed that the daily use of antiseptic baths (0.005% NaOCl) led to an improvement of all parameters both primary (Investigator's Global Assessment, IGA; Eczema Area and Severity Index, EASI; Body Surface Area, BSA) and secondary scores (Visual Analog Scale, VAS, for pruritus, Family Dermatology Life Quality Index, Patient Satisfaction Questionnaire for Problem Areas) [80]. At 2 weeks of treatment, 32/50 (64%) of *S. aureus-positive* patients were still colonized. The limited reduction in S. aureus colonization, despite the clinical improvement, suggests that NaOCl may have positive effects other than simple antimicrobial actions. Indeed, Leung et al. [81] demonstrated that NaOCl has a direct anti-inflammatory effect, by suppressing Nuclear Factor kappa-light-chain-enhancer of activated B cells (NFκB) signaling in cultured keratinocytes and reducing the severity of radiation dermatitis in mouse skin. Bleach baths down-regulate the mitogen-activated protein kinase (MAPK) and NF-kB pathways, leading to decreased production of proinflammatory cytokines (IL-1A, IL-6, TNF, IL-4, IL-13, TARC ) and pro-pruritogenic mediators (TSLP) [82]. Recently, it has been shown that at a concentration of 0.01-0.16%, NaOCl has an antimicrobial and antibiofilm effect against *S. aureus* [83] and improves the thickness and proliferation of the epidermis, without affecting the skin microbiota. Its use at higher concentrations has shown a significant decrease in the severity of AD [61].

Therefore, in individuals suffering from moderate to severe AD with a tendency to skin colonization by *S. aureus*, in addition to suitable topical anti-inflammatory therapy and careful personal and family hygiene, decolonization with bleach baths 2 times a week is recommended.

Potassium permanganate has also proved effective, although its use is limited by the pigmenting action (materials it comes into contact with and the skin) and the fact that in some countries, such as in Italy, it is not readily available.

### Antiviral therapy

#### Eczema herpeticum

Kaposi varicelliform eruption (eczema herpeticum, EH) is the name given to a distinct rash caused by the herpes simplex virus (HSV) and some other viruses that infect people with pre-existing dermatosis, most often AD. In general, the term EH is used to define acute and disseminated viral infection caused by HSV type 1, or more rarely type 2. EH represents a potentially serious complication of AD that usually occurs at the first herpetic infection, but it can also complicate relapses. Clinically it manifests itself with vesicles that evolve into blackish hemorrhagic crusts of a few millimeters, located mostly in the sites of AD and sometimes disseminated. Fever and lymphadenopathy are associated. In severe cases, viremia can cause complications such as keratoconjunctivitis, meningitis, and encephalitis that can lead to death. Bacterial superinfection of the lesions is also possible. Relapses are reported in 13-16% of cases [84].

The conditions favoring the onset of EH are severe, inadequately treated and early-onset AD, high levels of total IgE and peripheral eosinophils, filaggrin deficiency, and other concomitant atopic diseases [85].

However, since EH affects only 3% of subjects with AD [86], it has been hypothesized that atopic skin deficiency of plasmacytoid dendritic cells, antimicrobial peptide deficiency (e.g. cathelicidin LL37), and increased HSV receptor (nectin-1) expression, are the main pathogenetic factors of the disseminaon of viral infection. In infants, in whom HSV-1 generally causes the first herpetic infection, EH can be particularly serious as the local skin infection can progress to serious complications, including keratoconjunctivitis, encephalitis and septic shock.

For patients with extensive skin involvement, with signs of systemic disease and for those less than 1 year of age, systemic acyclovir therapy should be promptly given to shorten the course of the disease and avoid complications. In mild forms, aciclovir 400 mg is administered orally 5 times a day for 5-10 days, depending on the clinical course. Intravenous administration of aciclovir involves a dose of 5-10 mg/kg body weight, 3 times a day (or for children under 12 years 750 mg / m2 body surface area, 3 times a day) for 7 days until clinical recovery. Once clinical improvement has been achieved, acyclovir therapy can be continued and completed using oral formulation. As an alternative to aciclovir, derivatives such as valaciclovir (off label, at a dose of 20 mg/kg dose twice a day, max 1000 mg/dose, for 5-7 days), famciclovir, or, in more resistant cases, can be used. foscarnet.

For children with recurrent EH, a prophylactic administration cycle of oral aciclovir, 20 mg/kg/dose, twice a day, for 6 months (max 12 months) can be used to suppress relapses. In this case, electrolytes, kidney function, and white blood cell count will need to be monitored regularly [86].

If the AD is difficult to treat, TCSs can be continued during systemic acyclovir therapy without affecting the clearance of the viral infection. On the other hand, the use of TCIs during active infection is contraindicated [87].

#### Molluscum contagiosum

Defects of the skin barrier also predispose to the occurrence of *molluscum contagiosum* (MC) while long-term scratching leads to diffusion by autoinoculation.

The characteristic lesions of the MC present as pearly colored papules, 1 to 5 mm in diameter, with a typical central umbilication. The clinical appearance of MC is generally diagnostic and biopsy is indicated only in rare doubtful cases. It is not possible to carry out viral cultures of skin lesions, however, it is possible to detect the presence of viral DNA with molecular biology techniques in such lesions.

MC tends to resolve spontaneously. If the injuries cause discomfort, active treatment is possible. In the literature, it is reported that, from 2 years upwards, topical potassium hydroxide (KOH), at a concentration of 5% and 10%, can be applied at home in patients with single MC or with lesions limited to some areas of the body or extremities. Both concentrations are also suitable for the treatment of patients with a high number of lesions. In general, procedures that cause intense pain or are associated with a significant risk of scarring, such as curettage, cryotherapy, salicylic acid, imiquimod, should be avoided. A recent randomized placebo-controlled study also reported the efficacy of using cantharidin (not on the market in Italy) for the treatment of pediatric CD [88].

*Eczema molluscatum* [89] appears as an itchy eczematous eruption of the skin, diffuse or nummular, surrounding the MC. In these cases, the use of TCSs is recommended as it reduces itching and therefore the risk of spreading the virus from self-injection secondary to scratching. The treatment with TCIs can also be continued during *eczema molluscatum* infection.

#### Eczema coxsackium

Hands foot and mouth disease, caused by *coxsackievirus* A6, can present with atypical manifestations characterized by vesicular lesions, sometimes bullous, which in addition to the classic sites (hands, feet, and oral cavity) can involve large skin areas up to widespread forms, sometimes with hemorrhagic or purpuric appearance. These include *eczema coxsackium* (EC) which has similarities with EH since they develop on areas of pre-existing eczema [90, 91]. The blisters are relatively monomorphic and can be painful but generally not itchy. Blisters are more common in babies younger than one year of age than in older babies, who have blisters instead. Any site can be affected, even healthy skin, but hands, feet, face, trunk, and buttocks/groin are usually involved, preferably in areas already affected by AD. Oral ulcers, fever, and oropharyngeal pain may be present. EC resolves on its own, usually does not require hospitalization, and its management follows the standard of AD treatment, with continued skincare and anti-inflammatory treatments based on TCSs and bandages [92].

### Antifungal therapy

The role of *Malassezia spp* has been considered in the pathogenesis of a clinical variant of AD that presents in adolescence or adulthood with eczematous lesions of the head, neck (head and neck dermatitis) and upper trunk associated with intense pruritus and resistance to therapy with TCSs and/or TCIs [93–96].

*Malassezia spp* has been hypothesized to be the cause of this particular form of dermatitis. *Malassezia furfur* is a family of yeasts, mainly lipophilic, which normally colonize human skin from puberty, especially in the head and neck area and in the intertriginous folds. The close contact between *Malassezia spp* and the cutaneous immune system induces both a humoral and cell-mediated immune response. Specific IgE and positivity of the prick tests against specific antigens of *Malassezia spp* and sometimes also of the specific patch test have been demonstrated [93–96].

Treatment with itraconazole at (200 mg/day) was found to be effective. The duration of treatment is variable, from one week to a month, and is sometimes continued with 1 dose per week for several weeks. TCSs can be associated locally. Alternatively, fluconazole can be used. Cyclopyroxolamine is also indicated as a topical therapy in the treatment of "head and neck" AD [95–97].

Candida albicans species, frequently found in both healthy and damaged skin, may play a role in exacerbating skin lesions [98, 99].

The treatment consists of the application of topical azoles (clotrimazole 1% or miconazole 2% twice a day for 1–2 weeks); in refractory cases, a systemic azole such as single dose fluconazole is used.

The role of various dermatophytes (e.g. *Trichophyton Epidermophyton* and *Specie Microsporum*) in the pathology of AD is not yet fully understood but may be suspected in patients resistant to standard therapies (e.g., tinea incognita, variant of modified skin dermatophyte infection. incorrect use of a topical or systemic steroid). Topical antifungal drugs e.g. azoles, allylamines, butenafine, ciclopirox, and tolnaftate once or twice a day for 1–3 weeks are indicated for therapy.

In recalcitrant AD with dermatophyte infection, systemic treatment with azoles (200 mg/day of itraconazole) is used [100].

### New topical therapies: topical phosphodiesterase 4 inhibitors and jak kinase inhibitors

In recent years, the improved knowledge of the complex immunological mechanisms underlying AD, together with the progress of the pharmaceutical sector, is profoundly changing the therapeutic approach and has allowed the development of molecules capable of interfering with various intracellular pathways. In this regard, phosphodiesterase-4 (PDE4i) inhibitors and Janus kinase (JAKi) inhibitors are gaining increasing interest [101].

Crisaborole is the only topical PDE4 inhibitor to be approved in the United States by the FDA for the topical treatment of mild and moderate AD from three months of age. On March 27, 2020, the European Medicine Agency (EMA) authorized its marketing also in Europe, in patients with AD from two years of age. Crisaborole is a non-steroidal molecule capable of selectively inhibiting PDE4, resulting in an increase in intracellular levels of cyclic adenosine monophosphate (AMP) and preventing the release of inflammatory mediators, such as interleukins IL-2, IL-4 and IL- 5, tumor necrosis factor-alpha (TNF-α) and IFNγ, thus improving the protective function of the skin barrier [101, 102]. The demonstration of the efficacy of crisaborole is based on some important randomized trials, including the one conducted double-blind by Paller et al. [103] on 1,522 patients aged between 2 and 79 years, suffering from mild-moderate AD. Significant and more rapid improvement in pruritus and clinical signs of lesions was documented in the 960 patients treated with crisaborole who completed the study, compared with 438 who used the vehicle alone. Among the most common side effects were reactions at the application site, such as tingling and burning. However, the high cost of this drug currently limits its use to patients who cannot use TCSs and TCIs.

JAKi is a promising new class of topical drugs currently under study. Janus kinases (JAKs) are a family of intracellular tyrosine kinases that transduce signals mediated by cytokines and growth factors [104–106]. The results obtained from the phase I and II studies are extremely encouraging, as most patients quickly reach the primary outcome of the study, with a high safety profile. In these studies, there is also a rapid improvement in itching, which patients report easing from the first day of application of this drug.

Delgocitinib 0.5% (Corectim ®) ointment is the only JAKi that received approval in Japan in January 2020 for the treatment of AD in adults. It is a non-selective inhibitor of JAK, able to inhibit JAK1, JAK2, JAK3 and TYK2, which has been shown to improve the alterations at the level of the skin barrier, to favor the terminal differentiation of keratinocytes, to reduce and suppress itching induced by IL-13 [107]. In mouse models of AD, its topical application has been associated with an increase in NMF levels (natural moisturizing factors) and an improvement in skin inflammation and alterations in the skin barrier [107].

### Wet-wrap dressing

An important role in the pathogenesis of AD is represented by the increase in transepidermal water loss from the altered skin barrier. Wet-wrap therapy (WWT) is an ancient remedy that is considered an effective and safe second-line treatment, to be used in severe or refractory forms of AD, in patients older than 6 months of life [45, 108, 109]. This therapy must always be started and followed by experienced personnel.

There are two main methods for WWT. In the first method, after a short bath of 5-15 minutes with warm water, the skin is dried by dabbing without rubbing, then topical therapy is applied directly to the skin. Immediately afterward, the skin is bandaged with a double layer of gauze or tubular, of which the first layer (the internal one) is moistened with warm water, while the second (the external one) remains dry. Whenever possible, the humidification of the first layer is renewed every 2-3 hours during the day but not at night, with a steamer and warm water, after removing the dry layer of gauze or tubular. In the second method, a skin bandage is performed with two layers of gauze, the first moistened with diluted steroid or water directly on the injured skin and the second dry layer. In both methods, the use of an external state results in decreasing evaporation of water from the inner to the outer layer, thus resulting in cooling and prolongation of the moisturizing effect.

The TCSs most suitable for WWT are fluticasone propionate, methylprednisolone aceponate, mometasone furoate, hydrocortisone acetate and prednicarbate. TCSs are to be combined with an emollient with a hydrophilic base at a dilution of 10% (1 part of steroid and 9 parts of emollient) or 5% when treating the face [110–112].

Latex-free and washable viscose elastic bandages can also be used, which can be applied for 3-24 hours, although daytime bandages are always preferred despite the difficulty in being accepted by children. The duration of treatment varies from 2 to 14 days and generally, the best results are obtained during the first week. Recently, a study was published using a 100% nanopolyester fabric [113]. This less expensive, more acceptable, and the longer-lasting fabric has given excellent results.

WWT is an effective treatment thanks to its anti-inflammatory and cooling action [45, 108, 109]. Side effects vary according to the patient's age, the TCS used, the occlusion time, and the total duration of treatment [109] The most frequent adverse effects are related to poor acceptance and possible chills during the application of the wet layer. Bacterial and/or viral over-infections are also described. The most fearful side effect is the transient increase in cortisol levels due to systemic absorption of the TCSs used. Therefore, the WWT requires specialized personnel and can only be carried out at the patients' homes if they receive adequate and specific training.

### Special fabrics

Clothes, or rather fabrics in direct contact with the skin, can play an important role in patients with AD. It is widely demonstrated that some fabrics can be irritating factors (wool) or cause allergic contact dermatitis (colored fibers). On the contrary, some fabrics can have a protective role, by constituting a defensive barrier against exogenous irritants and favoring the formation of the physiological skin microbiome through antibacterial activities.

The ideal tissue for the skin of the child with AD should reduce transepidermal water loss, promote hydration, limit inflammation and itching. A fabric, therefore, made up of smooth fibers with a small diameter (the diameter of the fibers seems to be correlated with the ability to evoke itching) [114] non-occlusive and with antimicrobial capabilities that persist after several washing cycles. Occlusive fabrics such as polyester or nylon temporarily promote the trapping of water in the skin but when removed, they cause significant evaporation and transepidermal water loss.

The fibers used first for better control of AD are cotton and silk. Subsequently, natural cellulose-based fibers, such as Lyocell, were built. Recently, fibers with associated silver, quaternary ammonium, or other antibacterials have been developed to obtain an antibacterial action, in particular against S. aureus. The latter products are those that have been particularly studied in recent years. Table 6 lists the various fibers currently on the market.

**Table 6:** Characteristics of the main fabrics on the market

**Fabric type**	**Features**
Sea Cell Active fibers® Smart Fiber AG, Thuringia, Germany^a^	Made using Lyocell, dried algae are crushed, ground, and incorporated into cellulose fiber. The antibacterial effect is obtained through the activation of metal ions.
SkinDoctor® Ventex Co., Ltd., Korea	It is a silver-associated cellulose fabric made with algae, with a moisture control system. To produce the fabric, a semi-permanent antibacterial (titanium dioxide-silver) is applied to the regenerated rayon (Lyocell; Lenzing AG, Lenzing, Austria). This rayon represents 60% of the final fabric and the remaining 40% is made up of polyester.
Skintoskin® New Textiles, Ltd, London	It is a fabric of cellulose fibers with algae enriched with silver ions.
Chitosan	Chitosan is a product of the waste from the crustacean food industry. It is a biopolymer with biological, physiological, and pharmacological properties, such as biodegradability, non-toxicity, and strong antibacterial activity against both Gram-positive and Gram-negative bacteria thanks to the combined bactericidal and bacteriostatic action.
MICROAIR DermaSilk ® (AlPreTec Srl, San Donà di Piave, Italy^a^	Fabric is made of 100% silk fibroin, an animal protein composed of the same amino acids (glycine, alanine, serine, etc.) that form the stratum corneum, with added ammonium quaternary.
DreamSkin TM (DreamSkin Health Ltd, Hatfield, UK)	Fabric made with silk fiber finished with DreamSkin® polymer and a zinc-based antibacterial. It is based on the same technology used for contact lenses.
Padycare® Texamed GmbH	It is made of silver-coated polyamide fibers.
Binamed® Binamed Moll GmbH	It consists of two different yarns, the micro modal fiber, and the silver thread.

^a^Available in Italy

Two clinical trials, one open-label [115] and one double-blind controlled [116] have documented the efficacy of silver-coated cotton fabric in reducing SCORAD and *S. aureus* colonies in patients with moderate AD. The use of a seaweed-based fabric with associated silver (Sea Cell Active fibers®, Smart Fiber AG, Thuringia, Germany) resulted in an improvement in transepidermal water loss [117] and a reduction in the number of *S. aureus* colonies in patients with mild-moderate AD [118]. A randomized controlled study with a silver-associated cellulose-based tissue (SkinDoctor® Ventex Co., Ltd., Korea) in patients with mild to moderate AD also described an improvement in SCORAD, a reduction in transepidermal water loss and a reduction of *S. aureus* colonies, compared to cotton tissue [119]. A double-blind randomized controlled study with a cellulose-based fabric with an associated silver (Skintoskin® New Textiles, Ltd, London) observed an improvement in SCORAD, sleep quality and itching as early as 7 days, with a lasting effect for 90 days, compared to cotton fabrics [120]. The possible percutaneous absorption of silver, which is greater on damaged skin, should be considered. However, a recent study showed no increase in silver in urine in the group who wore silver clothing for at least 8 hours a day for 5 days [121] Chitosan-based fabrics (a biopolymer derived from chitin) also show antibacterial activity. A double-blind controlled trial in adolescents and adults who wore cotton pajamas with chitosan, showed an improvement in SCORAD with a reduction in the use of topical therapies compared to the group who wore only cotton [122].

Several but similar randomized controlled trials have compared fabrics enriched with quaternary ammonium (MICROAIR DermaSilk ® AlPreTec Srl, San Donà di Piave, Italy) vs untreated cotton or silk fabrics, in pediatric patients with mild to moderate AD [123–127]. These studies have shown encouraging results, reporting improved clinical AD scores and reduced exacerbations associated with the use of such fabrics compared to the control tissue.

A randomized controlled trial, conducted on 300 children with moderate-severe AD, compared the clinical efficacy of two silk garments made from antimicrobially protected knitted sericin-free silk, Dermasilk TM and DreamSkin TM (DreamSkin Health Ltd, Hatfield, UK), with standard therapy, and did not show significant clinical improvement after 6 months [128]. However, it should be noted that in this study, most of the children presented a severe clinical picture.

Recently, a study evaluated the persistence of silver and quaternary ammonium in fabrics after several washing cycles [129]. It should be noted that the fabrics in which silver is added do not behave in the same way. Only Padycare® and Binamed® show persistence of silver even after 150 cycles. Instead, less relevant results are found with other fabrics added with silver. Even less persistent is the quaternary ammonium, which is no longer detected after 30 washing cycles. While there is a rationale in the possible use of fabrics and/or garments that have structural characteristics to counteract the inflammation of AD, there is currently insufficient robust data to suggest their routine use, as claimed in a recent review of evidence-based literature [130]. New controlled studies will be able to provide more precise indications in this regard.

### Oral corticosteroids

Oral corticosteroids act rapidly on acute lesions of AD by reducing inflammation. However, despite their widespread use, few randomized controlled clinical trials have been performed to confirm their superiority compared to other drugs [131, 132]. Besides, they have a limited role in itching and pain [133].

Despite their effectiveness, the risk of systemic side effects strongly limits their use. When used in the long term, they can lead to suppression of the hypothalamic-pituitary-adrenal axis, immunosuppression, hypertension, weight gain, osteoporosis, and stature growth retardation in children [131, 134]. Upon discontinuation, a significant rebound of acute AD has frequently been described [131, 132]. Therefore, the use of oral corticosteroids should only be recommended in exceptional cases, for a limited time (e.g. in case of major exacerbations in patients with severe AD), and with close monitoring for possible side effects, especially in pediatric patients [45, 132]. Oral corticosteroids could be used for a 1-2 week cycle according to the following scheme: methylprednisolone at a dose of 0.5 mg/kg/day for 1-2 weeks, tapering the drug in about a month [45, 135].

### Cyclosporin A

Cyclosporin A (CsA) is an oral immunosuppressive agent belonging to the calcineurin inhibitor family, used in the prevention of organ transplant rejection and to treat various inflammatory skin diseases, including psoriasis and AD [136].

CsA acquires activity upon binding with cytoplasmic proteins known as cyclophilins and this complex competitively binds and inhibits calcineurin. This reduces the transcription of genes encoding IL-2, TNF ‐ α, IL-3, IL-4, INF-γ, GM-CSF, and leads to the reduction of lymphocytes T [137]. In patients with AD, CsA can reduce eosinophil counts, E-selectin, and soluble CD30 levels and correct the Th1 / Th2 imbalance [138]. The dosage range is between 2.5-5 mg/kg/day.

Based on a systematic review of 34 randomized clinical trials [139] and a meta-analysis and review of 15 randomized trials [140], CsA is currently recommended as a conventional therapeutic option in the short-term treatment of moderate-to-severe AD in adults. The majority of patients experience a significant reduction in disease activity within 2-6 weeks of starting therapy and its estimated efficacy, expressed as a reduction in AD severity scores, is between 53 and 95% [139].

In the first clinical trial on the use of CsA in pediatric AD, 27 children aged between 2 and 16 years of age were treated with CsA for 6 weeks at a dose of 5 mg/kg/day [141, 142]. In this open study with short-term observation, a significant improvement was observed in all measures of disease activity in the majority of treated patients. Treatment was well tolerated and there were no significant changes in serum creatinine and/or blood pressure. However, after discontinuation of the drug, the majority of patients experienced a relapse of AD within a few weeks.

In a subsequent prospective, open, parallel-group, multicenter study, 40 children aged 2 to 16 years with severe AD unresponsive to TCSs therapy, were randomized to receive either CsA in a continuous long-term mode (up to 12 months) or an intermittent short-term schedule (multiple cycles of 12 weeks) [143]. The starting and maximum dose of CsA for all patients in the 2 groups was 5 mg/kg/day. CsA was effective with both therapeutic regimens. The continuous therapy scheme showed a more consistent improvement, although the short-term regimen proved also to induce a prolonged remission of AD in some patients, with a reduction in the cumulative exposure to CsA. Therefore, the possibility of using tailored dosages on an individual basis was suggested [143].

CsA has also been compared to methotrexate in a randomized clinical trial involving 40 children aged 8 to 14 years with severe and TCSs refractory AD and poor compliance or poor response to phototherapy [144]. The drugs were administered in low doses, 2.5 mg/kg/day for CsA and 7.5 mg/week for methotrexate, respectively. Both groups of patients showed a statistically comparable reduction in SCORAD. CsA showed greater rapidity of action (2–3 weeks), but also greater relapse of AD (mean 14 weeks) after discontinuation. A randomized, controlled trial on the efficacy and safety of methotrexate versus cyclosporine in severe AD in children is ongoing [145].

Currently, the use of CsA is off-label in children and adolescents, while it is approved by the European Medicines Agency (EMA) for severe AD in adults [146]. However, its use is widely accepted in children with moderate-to-severe AD [147]. In such cases, most patients exhibit a rapid and good to excellent improvement in AD, with a very low incidence of serious side effects [148]. A 2013 survey conducted by the European Treatment of Severe Atopic Eczema in Children Taskforce (TREAT) indicated CsA as the first-line systemic therapy for severe AD in the pediatric age [149].

CsA is usually administered with intermittent treatment regimens lasting up to 12 months. A break of 2 weeks before and again 4-6 weeks after vaccination is suggested, although there is no evidence to support such management [146]. Potential adverse events include infections, nephrotoxicity, hypertension, tremor, hypertrichosis, headache, gingival hyperplasia, and increased risk of skin cancer and/or lymphoma. Evaluation of side effects should include physical examination, blood pressure measurement, regular monitoring of complete blood counts, blood urea nitrogen, creatinine, electrolytes, magnesium, uric acid, bilirubin, cholesterol, triglycerides and urinalysis. Caution is advised in patients taking other medications due to the many possible drug interactions [15, 150].

European guidelines suggest that CsA should be administered in patients with SCORAD index> 50 and/or with persistent AD [15]. A very recent systematic review has shown that CsA and dupilumab are equally effective, compared to placebo, in inducing remission of clinical signs of AD and are more effective than methotrexate and azathioprine in the short term [150].

### Methotrexate

Methotrexate (MTX) is an antimetabolite that inhibits the synthesis of folate, thereby blocking the synthesis of purines, DNA and RNA. Its therapeutic action is probably linked to the inhibition of the function of T lymphocytes [151]. In recent years, numerous studies have shown the efficacy of MTX in adults with AD in the absence of serious adverse events [134, 152]. A randomized study comparing MTX vs azathioprine showed similar effects in severe AD [153]. In a randomized, controlled pediatric study comparing MTX vs CsA, the MTX-treated group achieved a similar reduction in SCORAD to that seen in the CsA-treated group [144]. A retrospective study of children undergoing MTX treatment for severe AD demonstrated that MTX is an effective and safe drug with long-term lasting therapeutic effects [154]. A randomized and controlled study on the efficacy, safety and cost/benefit ratio of MTX vs CsA in the treatment of severe AD in children is currently underway [145].

In a retrospective study of 55 pediatric patients, the clinical severity score of AD significantly improved in most patients, demonstrating that MTX is an important option for long-term control in severe AD with a favorable adverse effect profile and costs [155].

However, in a recent systematic review and meta-analysis on adult patients, CsA and dupilumab showed greater short-term efficacy than MTX and azathioprine in patients with severe AD [150].

Safety data for MTX are derived from studies of other diseases treated with MTX. Stomatitis, nausea and vomiting are reversible adverse effects with dose reduction and/or discontinuation of treatment. Liver toxicity and teratogenicity, in both sexes, are the major side effects. The blood count and liver enzymes must therefore be monitored during treatment. Screening for hepatitis B and C and chest x-rays should be performed before starting treatment with MTX. Folic acid supplementation is always recommended during treatment with MTX to reduce hematological and gastrointestinal toxicity.

MTX appears to have a slow response (8-12 weeks) but is maintained over the long term. The recommended therapeutic dose in children is between 0.2 and 0.5 mg/kg/week (maximum 25 mg/week) and the duration of treatment is 10–16 weeks [45]. The drug can be increased by 2.5–5 mg/week and decreased by 2.5 mg/week, up to the lowest effective dose [15]. In conclusion, the off-label use of MTX can be considered as a second-line systemic treatment option, in particular when prolonged treatment is needed.

### Azathioprine

Azathioprine is a purine analog that has immunosuppressive activity by inhibiting DNA production, therefore affecting cells with high proliferation rates, such as T and B lymphocytes during the inflammatory disease phases [139, 156–158].

Azathioprine can be considered a second-choice therapy for moderate-severe AD in adults when CsA has not shown efficacy or has given side effects or is contraindicated [139, 156]. Azathioprine has lower efficacy than CsA, but comparable to that of methotrexate. The recommended dosage is 2-3 mg/kg/day and it generally takes at least 1-2 months to manifest the beneficial effects [139, 156]. The low thiopurine-methyltransferase activity has been associated with increased myelotoxicity, but at-risk patients can be identified before starting azathioprine therapy by testing the activity of this enzyme. If this test is not available, one can start with a low dose of azathioprine and monitor blood tests (i.e. complete blood count, liver, and kidney function) after 7-15 days to gradually increase the dose. It is advisable to continue to monitor blood tests regularly throughout azathioprine therapy [139]. Because of its slowness of action, azathioprine can initially be used in association with TCSs. The most reported side effects associated with the use of azathioprine are gastrointestinal symptoms, myalgia, fever, skin reactions, headache, lymphopenia, and neutropenia.

In conclusion, azathioprine is a drug rarely used in the treatment of AD in adults and even less in the pediatric age, in which azathioprine is to be considered an off-label therapy [139, 156–158].

### Mycophenolate mofetil

Mycophenolate mofetil is rarely used in the treatment of AD. It is an immunosuppressive drug that acts by inhibiting the enzyme inosine monophosphate dehydrogenase, thus blocking the synthesis of purines, and reducing the proliferation of T lymphocytes [159–162].

Mycophenolate mofetil is proposed as a third-choice treatment in patients with severe AD in whom cyclosporine has not shown efficacy, has produced side effects and/or is contraindicated [159–162].

It can be prescribed at an initial dosage of 10-40 mg/kg/day, increasing the dosage by 500 mg every 2-4 weeks until the effective dose of 20-50 mg/kg/day is reached. Mycophenolate mofetil appears to offer a good efficacy and safety profile in the treatment of adult patients with severe AD and the most common adverse effects are gastrointestinal disturbances (nausea, diarrhea, and abdominal pain). It can be teratogenic in both sexes [159–162].

### Antihistamines

Oral antihistamines have always been included in guidelines for the treatment of AD, although their usefulness is much debated [163, 164]. Itching is the most common feature of AD and the one that most affects patients' quality of life. Itching triggers scratching which perpetuates skin inflammation through the release of various mediators, such as thymic stromal lymphopoietin (TSLP), thus feeding the "itch-scratching" cycle [134–165]. Through a competitive mechanism on H1 receptors, oral antihistamines act by blocking the effects produced by histamine (vasodilation, itching). Most guidelines recognize a possible role of first-generation sedating H1 antihistamines in the treatment, for short periods, of sleep disturbances associated with AD pruritus, due to their sedative effect [134, 166–168]. Some authors recognize a rationale in the use of oral antihistamines only in patients with concomitant AD and allergic rhino-conjunctivitis [134, 149, 169].

In 2015, the EMA has issued a warning on the safety of the first-generation H1 antihistamines under 2 years, in particular for hydroxyzine, due to the possible undesirable effects on the electrical activity of the heart (low but defined risk of QT prolongation and torsades de pointes) [170]

In 2018, a Cochrane review assessed 25 clinical trials, including 8 studies conducted in pediatric populations, to investigate the efficacy of oral second-generation H1 antihistamine as an adjunct to topical therapy in AD [171]. While reporting that the evidence in this regard was qualitatively limited, this review did not show a consistent efficacy of the “add-on” therapy with second-generation H1 antihistamine on AD. No clinical efficacy emerged for cetirizine and loratadine compared to placebo, although it confirmed their safety in use [172].

The most common side effects of antihistamine therapy are excessive and unwanted sedation (even for non-sedating antihistamines), and anticholinergic symptoms (e.g. dry mouth, blurred vision, tachycardia [165]. Furthermore, since first-generation antihistamines with sedative properties can influence daytime wakefulness, particular attention is required in the dosage and administration schedule, especially in school-age children due to possible negative interference on school performance, and in adolescents who are allowed to drive motorcycles and automobiles [172–174].

Lastly, it should be noted that the use of topical antihistamines is not recommended for the risk of absorption and systemic toxicity (e.g. reported with diphenhydramine) [175] and for the possibility of promoting the onset of allergic contact dermatitis [15, 176].

### Probiotics

The possible use of probiotics in the prevention and treatment of AD has recently been the subject of numerous studies. Recent data show that patients with AD have a dysbiosis of the intestinal microbiota, with a reduction in Bifidobacterium spp and an increase in Staphylococcus spp, *Escherichia coli* and *Clostridium difficile*, compared to healthy individuals [177]. This dysbiosis is associated with a reduction in the activity of regulatory T lymphocytes, which favors the increase in intestinal permeability and loss of immunological tolerance [178, 179]. These observations support the hypothesis that a specific composition of the intestinal microbiota may modify the imbalance towards Th2 responses observed in AD, thus favoring an immune-regulatory response [180, 181].

In recent decades, several clinical trials have examined the possible efficacy of probiotics in treating AD, often with conflicting results [180, 182]. A recent Cochrane review of 39 randomized controlled trials analyzed data from 2,599 individuals with AD who took probiotics of the *Lactobacillus* or *Bifidobacterium* species, alone or in combination with other probiotics, with or without supplementation with prebiotics. Different doses and concentrations were used in the trials examined and the duration of active therapy ranged from 6 weeks to 3 months, thus resulting in an extreme heterogeneity of results. Overall, the comparison between patients treated with probiotics versus untreated ones revealed little or no efficacy of the therapy on skin symptoms, as reported by the patient and/or parents, and on the severity of the skin condition, documented by the investigators. Furthermore, there was also no evidence of benefit in terms of quality of life. Therefore, the authors conclude that, according to the available evidence, the use of probiotics for the treatment of AD is not an evidence-based approach [183]. Further studies are needed to clarify and define which bacterial strains are effective, the doses, the schedule and the duration of therapy.

Finally, in recent years, the in vitro demonstration that some strains of probiotics can exert an anti-inflammatory, antimicrobial and barrier activity has led to the hypothesis of possible topical use of both probiotics and their derivatives (e.g. bacterial lysates) in the treatment of AD [184–186]. Recently, the use of a topical emollient containing lysates of the Gram-negative bacterium *Vitreoscilla filiformis* grown in thermal water (LRP-TSW), has been shown to improve SCORAD and normalize the microbial flora with reduction of *S. aureus* in patients with AD [187, 188], *Lactobacillus reuteri* is another highly studied probiotic strain, which has shown a good tolerability and safety profile and could therefore be a promising topical therapy [189].

### Phototherapy

The definition of phototherapy comes from the Greek "light therapy" and refers to a technique that uses the beneficial effect of light waves in various pathological conditions. The technique can be performed using different artificial light sources, which include broadband UVA (315-400 nm) and oral psoralens (PUVA), UVA and topical psoralens (bath-PUVA), UVA-1 or long UVA (340–400 nm), broad‐spectrum UVB (280–315 nm) and narrowband (NB‐) UVB (311–313 nm) [45].

Phototherapy is indicated as a second-choice therapy for moderate and/or relapsing forms of AD not responsive to TCSs and/or TCIs [45]. This treatment, used in both adults and children (typically> 12 years of age), induces improvement in skin lesions, pruritus, and sleep (Strength of recommendation B and level II of evidence [45, 190–193].

PUVA phototherapy is rarely used, due to the important side effects such as cataracts, nausea, headache, itching, skin hyperpigmentation and, in the long term, also the increased carcinogenic risk [45, 190–192,193].

UVA1 phototherapy is effective in the acute phase of AD because it favors T-lymphocyte apoptosis, lower expression of pro-inflammatory cytokines, including IL-5, IL-13, IL-31, and reduction of dendritic cells. Currently, medium doses (65 J/cm2) are preferred to high doses (130 J/cm2), which produce excessive heat and intense sweating. The use of the so-called "UVA1 cold light", which uses lamps that filter infrared rays, has shown a better tolerance than traditional UVA-1 or UVA/UVB. The therapeutic scheme of UVA1 phototherapy includes 3-5 sessions per week for 3-8 weeks with a maximum dose of 80 J/cm2. The duration of the single session can vary from 10 to 60 minutes. Although effective, UVA1 therapy is only available in a few centers, and its use is limited by possible long-term side effects, such as photo-damage and carcinogenic risk. Therefore, UVA1 phototherapy is currently only recommended for adult AD and its use in pediatric age is rare [45].

NB-UVB or UVBTL01 phototherapy is indicated for chronic moderate forms of AD due to the limited penetration of the UVB used [45, 194]. The treatment has a good anti-inflammatory activity, reduces the colonization of *S. aureus*, promotes the improvement of the skin barrier function, and the thickening of the stratum corneum, with a consequently increased resistance to environmental irritants [9, 194].The starting dose is calculated from the minimum erythematogenic dose or according to the phototype according to Fitzpatrick. The number of weekly sessions is variable as well as the duration of the treatment, as it can be used in short cycles until remission or in longer cycles as maintenance therapy. In general, the scheme includes 6-12 weeks of treatment, with 2-3 sessions/week with subsequent reduction to 1-2 sessions/week once the remission of the skin lesions is achieved. A 50% improvement in SCORAD with 3 sessions per week of NB-UVB for 12 weeks has been reported and the result may persist for up to 6 months beyond the end of treatment [9, 194].

In the pediatric age, NB-UVB phototherapy is considered the best therapeutic option for efficacy, good tolerability, low risk, and availability. Sometimes NB-UVB phototherapy can cause excessive heat and increase sweating with the possible flare-up of the AD. Air-conditioned NB-UVB regimens improve tolerability and have been shown to be more effective. The most common acute side effects of NB-UVB phototherapy are erythema, sunburn, xerosis. NB-UVB phototherapy can be used in monotherapy or combination with emollients and TCSs. Due to the possible carcinogenic risk, it is recommended to avoid combination therapy with TCIs [45]. In adults with psoriasis, NB-UVB phototherapy was performed in addition to biologics and this combination therapy resulted in better and faster responses than monotherapy [195].

The safety of long-term NB-UVB treatment has been well documented in psoriasis studies, however, short courses are recommended in children [154, 190]. The main limitations of NB-UVB phototherapy are the only hospital availability and the poor response of some sites, such as the scalp, folds, and eyelids which must be covered with protective goggles. Age-specific factors, such as fear of devices, poor compliance of the pediatric patient, and concerns of caregivers, should not be underestimated in the treatment decision. Therefore, it is necessary to be cautious and not to use phototherapy in prepubertal age. Subjects with light skin, phototype I and II, require additional attention as they tend to easily burn [154, 190]. It should be noted that the European Task Force on AD does not recommend the use of phototherapy in children under the age of 12 due to potential long-term side effects, although it is not contraindicated in children and properly selected cases [45].

Home phototherapy is little used in AD and the data available refer to the treatment of psoriasis. Cameron et al. [196] reported observations from a 13-year follow-up to a UK home service with NB-UVB and concluded that home phototherapy is just as effective as hospital phototherapy. However, home phototherapy requires motivated patients who adhere to the instructions and the supervision of an expert (dermatologist). Home phototherapy can be considered an option for patients in whom phototherapy cannot be used in conventional settings [197].

### Dupilumab

The main immunological mechanism in AD is an excessive type 2 inflammatory response [8, 9]. Eczematous skin lesions have a complex and varied inflammatory pattern but are dominantly characterized by the expression of Th2 CD4 + and innate type 2 lymphoid cells (ILC2) [45, 64]. The release of alarmins (DAMPs), determined primarily by the alteration of the skin barrier, activates the resident dendritic cells to promote a type 2 response with the release of IL-4 and IL-13 and other Type 2 cytokines, as chemokines related to thymic activation (TARC), TSLP and IL-33. IL-4 and IL-13 can block the production of some proteins that contribute to the integrity of the skin barrier. These two cytokines contribute to the differentiation of keratinocytes and down-regulate the production of filaggrin, loricrin and involucrin, adhesion molecules, antimicrobial peptides (beta-difensins and cathelicin LL-37) and ceramides, amplifying the skin barrier damage [198]. IL-4, IL-13, TSLP, and IL-33 contribute to pruritus, the predominant symptom of AD. The discovery of the IL-4Rα rceptor for IL-4 on afferent neurons and the control of pruritus itself through inhibition of the IL-4Rα receptor and JAK, has further reinforced the clinical relevance of the interaction between Type 2 immune response and neuronal pathways of pruritus [199].

Dupilumab is a human IgG4 monoclonal antibody, with molecular weight 147 Kda, directed against the alpha subunit of the IL-4 receptor (IL4Rα), an essential subunit for inducing IL-4 and IL-13 signaling. The biological function of IL-4 and IL-13 is mediated by the binding of two receptor subtypes (IL4R), which share IL-4Rα. The type I receptor, IL-4R, composed of the heterodimer IL-4Rα / gc, binds exclusively to IL-4 while the type II, composed of IL-4Rα / IL-13Rα1, binds both IL-4 and IL- 13 [199, 200].

The first studies on the clinical use of dupilumab in AD were performed in adults. Promising results have been reported in phase III, randomized, double-blind registration studies [201, 202] that evaluated 2119 patients aged 18 years or older with moderate to severe AD not adequately controlled by topical therapies (*SOLO 1: 671 patients; SOLO 2: 708 patients; CHRONOS 740 patients*). Based on the results observed in these studies, dupilumab was approved by the FDA in March 2017 and by the EMA in September 2017 for the treatment of adult patients with moderate to severe AD who are candidates for systemic therapy (Table 7).

**Table 7:** History of approvals of dupilumab for atopic dermatitis by international regulatory agencies

**Approval date**	**FDA**	**EMA**	**AIFA**
Adults with moderate to severe AD inadequately controlled	March 2017	September 2017	August 2018^a^
Adolescents 12-17 years, with moderate to severe AD inadequately controlled	March 2019	August 2019	November 2020^b^
Children 6-11 years, with moderate to severe AD inadequately controlled	May 2020	October 2020	January 2022^c^

^a^Adult patients with EASI score ≥ 24, for whom cyclosporine therapy is contraindicated, ineffective, or not tolerated. Reimbursement class: H, medicinal product subject to restrictive medical prescription, to be renewed from time to time, sold to the public on prescription from hospitals or dermatologist specialists (RNRL)

^b^Adolescents eligible for systemic therapy without prior use of cyclosporine; medicine subject to a limited medical prescription, to be renewed from time to time, sold to the public on prescription from hospitals or specialists - dermatologist, pulmonologist, allergist, otolaryngologist, immunologist and pediatrician (RNRL)

^c^From 20 December 2022 it is possible to use dupilumab in a reimbursable regime as an innovative non-oncological drug for the treatment of severe AD in children aged 6 to 11 eligible for systemic therapy who have an EASI score ≥24 or one of the following characteristics: localization in visible and / or sensitive areas; evaluation of pruritus with NRS ≥7 scale; quality of life assessment with CDLQI index ≥10

In 2020, a Phase 3 study was published in 251 adolescents (12-17 years of age with moderate-severe AD, not adequately controlled by topical therapies) which also demonstrated the efficacy and safety of dupilumab in this age group [203]. The treatment period was 16 weeks. Three groups were considered: a first group (85 patients) received placebo; a second group (82 patients) received subcutaneous dupilumab every 2 weeks with a weight-dependent dose, i.e. 200 mg if baseline weight was <60 kg [loading dose of 400 mg (43 patients)] and 300 mg if baseline weight ≥ 60 kg [loading dose of 600 mg(39 patients)]; the third group (84 patients) received dupilumab 300 mg every 4 weeks (600 mg loading dose) [203]. TCSs, TCIs, and crisaborole could be used as rescue therapy. Dupilumab showed rapid and significant efficacy in reducing signs and symptoms of AD, including pruritus, and in improving quality of life, even in patients who only achieved incomplete lesion remission. In particular, at week 16, the mean EASI improvement from baseline was approximately 66% compared to 24% achieved by placebo. In addition, 42% of patients on dupilumab achieved a ≥ 75% improvement in the EASI score (EASI-75), compared with 8% in the placebo group. Finally, 24% of patients on dupilumab achieved complete or near-complete remission compared to 2% on placebo, as measured by an IGA score of 0 or 1. Rescue therapy was primarily required in placebo-treated patients. The main side effects were seen in adolescents, i.e. allergic and infectious conjunctivitis and keratoconjunctivitis (10.8 and 9.8% respectively in treated every 4 or 2 weeks of cases versus 4.7% in placebo) and injection site reactions (6 and 8.5% versus 3.5% of placebo), were similar to those found in adults. Conversely, skin infections, observed in 20% of placebo patients, were found only in 13.3 and 11% of treated patients [203]. In conclusion, the efficacy and safety data of dupilumab in adolescents were consistent with those observed in adults (Table 8).

**Table 8:** Dupilumab in adolescents with moderate to severe dermatitis

Main clinical trials
Phase 2 open-label study R 668-AD1412
Pharmacokinetics, safety, efficacy in patients aged 6 to 17 years
Liberty ADOL
Phase 3 pilot monotherapy study R 668-AD 1526
Safety and efficacy in patients aged 12 to 17 years
Liberty AD PED OLE
Phase 3 study Open-Label Extension (OLE) R 668-AD 1434
Safety and efficacy in patients 6 months to <18 years

Dupilumab was therefore approved on March 11, 2019 by the FDA and on August 6, 2019 by the EMA for the treatment of adolescents with moderate to severe AD inadequately controlled with topical therapies or when such therapies are not advisable.

The “Redefine Atopic Dermatitis in Adolescent: an italian report” (RADAR) is a consensus project conducted on adolescents with AD [204]. The results of this project confirmed the high levels of efficacy and tolerability of dupilumab in this patient group, underlining the role of this biologic as a first-line systemic agent in the treatment of moderate to severe AD.

Even more recently, a randomized, double-blind, placebo-controlled phase 3 study (LIBERTY AD PEDS) was conducted in children aged 6 to 11 years with AD diagnosed more than 1 year prior to screening [205]. In total, 367 patients were randomized to be treated for 16 weeks either with dupilumab every two weeks (100 mg for weight 15-30 kg and 200 mg for weight> 30 kg), or placebo or dupilumab every four weeks (300 mg independently weight). Concomitant use of medium strength TCSs once daily was allowed in all groups. The primary endpoint was the achievement of IGA 0-1 at week 16 and the co-primary endpoint in the EU reference countries was EASI-75. Secondary endpoints were the percent changes in EASI and pruritus NRS scale from baseline to week 16. Both groups of patients treated with dupilumab + TCSs showed statistically significant improvement in signs and symptoms of AD, and in quality of life compared to patients treated with placebo + TCSs. Specifically, at week 16, the results showed total or near-total clearance of skin lesions (IGA 0 or 1) in 33 and 30% of patients treated with dupilumab every four weeks (300 mg) and every two weeks ( 100 mg or 200 mg) respectively, compared with 11% of patients treated with placebo (*p* <0.0001 and *p* = 0.0004, respectively). 70% of patients treated with dupilumab every four weeks and 67% of patients treated with dupilumab every two weeks achieved EASI-75, versus only 27% of patients treated with placebo (*p* <0.0001 in both cases). In addition, the biological drug showed significant relief from itching and improved anxiety and/or depression reported by both patients and their parents, and quality of life. The main side effects found in the study were: both allergic and infectious conjunctivitis and atopic keratoconjunctivitis (6.7 and 14.8% respectively in treated every 4 or 2 weeks of cases versus 4.2% in placebo), keratitis (observed in one patient in the dupilumab group every two weeks 100 mg or 200 mg) and injection site reactions (10 and 10.7% dupilumab group versus 5.8% for placebo). Conversely, skin infections, observed in 13.3% of patients on placebo, were found only in 5.8 and 8.2% of treated patients [205].

On May 26, 2020, the FDA approved dupilumab for the treatment of patients 6 years of age or older with moderate to severe AD whose disease is not adequately controlled with topical therapies or when such therapies are not advisable. Dupilumab can be used with or without TCSs. The EMA approved the use of dupilumab from 6 years of age on November 30, 2020, but only recently (January 29, 2022) the Italian Medicines Agency (AIFA) approved it for this age group (Table 7).

Table 9 reports the recommended posology of dupilumab for the treatment of pediatric AD [206].

**Table 9:** Recommended dose of dupulumab for the treatment of pediatric atopic dermatitis

Adolescents between the ages of 12 and 17		
*Bodyweight*	*Starting dose*	*Subsequent doses*
Less than 60 kg	400 mg (two 200 mg injections)	200 mg every 2 weeks
60 kg or more	600 mg (two 300 mg injections)	300 mg every 2 weeks
Children between the ages of 6 and 11		
*Bodyweight*	*Starting dose*	*Subsequent doses*
15 to less than 60 kg	300 mg (one 300 mg injection) on day 1, followed by 300 mg on day 15	300 mg every 4 weeks^a^, starting 4 weeks after the day 15 dose
60 kg or more	600 mg (two 300 mg injections)	300 mg every 2 weeks

^a^The dose may be increased to 200 mg every 2 weeks in patients weighing 15 kg to less than 60 kg based on the physician's assessment

Therapeutic efficacy is assessed after 16 weeks of treatment, which must be discontinued if there is no improvement. Live and live attenuated vaccines should not be administered concomitantly with dupilumab as safety and clinical efficacy have not been established. Immune responses to diphtheria-tetanus-acellular pertussis (DTPA) vaccine and meningococcal polysaccharide vaccine were evaluated. It is recommended that patients repeat immunizations with live and live attenuated vaccines, per current immunization guidelines, before treatment with dupilumab [206].

### New drugs

Recent advances in the knowledge of the underlying mechanisms of AD have contributed to the development of new systemic therapies, with high efficacy and safety profiles, which target specific molecules involved in the pathogenetic pathways of AD. Promising biological therapies with monoclonal antibodies targeting IL-31, IL-13, IL-22, JAK and other cytokines are under development. [207]. None of the drugs described below are currently prescribable for pediatric AD.

#### IL-31 inhibitors

IL-31 plays a key role in the pathophysiology of AD and pruritus. Nemolizumab is a selective monoclonal antibody for the IL-31 alpha receptor (IL-31Rα) which inhibits the signal of this interleukin [207]. In a recent phase 2b studies, nemolizumab administered for the treatment of moderate to severe AD in adult patients at a dose of 30 mg every 4 weeks demonstrated significant clinical improvement in AD, expressed by a significant reduction in both EASI and NRS-itch scores, compared to placebo [208]. The most common side effects reported were: exacerbation of dermatitis, nasopharyngitis, respiratory tract infections and increased creatinine levels.

#### IL-13 inhibitors

Lebrikizumab and tralokinumab are monoclonal antibodies that selectively inhibit IL-13.

Lebrikizumab t inhibits the activity of IL-13 by binding to the IL-13 receptor α1 (IL-13Rα1) [209]. In a phase 2 study, lebrikizumab administered in patients with moderate to severe AD at a dose of 250 mg every 2 weeks, demonstrated good efficacy results, expressed by a significant reduction in both the clinical severity of the disease (EASI and IGA scores) and the intensity of itching (NRS score), compared to placebo [210]. Lebrikizumab demonstrated a good safety profile and the most common side effects were related to injection site skin reactions.

Tralokinumab is an IgG4 monoclonal antibody that acts by selectively inhibiting the activity of IL-13, binding to both the α1 and the α2 receptor of IL-13 [207]. Tralokinumab, administered in patients with moderate-severe AD at a dose of 300 mg every 2 weeks, has shown significant efficacy results, leading to a significant reduction in both the clinical severity of the disease (expressed by EASI and IGA score) and the intensity of the pruritus (expressed by NRS score), compared to placebo [211]. Among its most common side effects are reported respiratory tract infections, headache and skin reactions at the injection site.

#### TSLP inhibitors

Tezepelumab is a monoclonal antibody that works by selectively inhibiting TSLP. It demonstrated good efficacy results, albeit not statistically significant (expressed as a 50% reduction in the EASI score from baseline), in the treatment of moderate-to-severe AD in adults, at the dose of 280 mg every 2 weeks, compared to placebo [212].

#### OX40 inhibitors

GBR 830 is a monoclonal antibody that acts by inhibiting the OX40 receptor in the Th2 pathway. It has shown good efficacy results in the treatment of moderate-severe AD with two IV administrations of 10 mg/kg every 4 weeks, inducing a significant reduction in the thickness of the epidermis and expression of specific mRNA-biomarkers and a clinical improvement, expressed by the reduction of EASI score, compared to placebo [213].

#### Inhibitors of IL-22

Fezakinumab is a monoclonal antibody that selectively inhibits the activity of IL-22, a cytokine that appears to play a key role in barrier dysfunction and epidermal hyper-proliferation [1]. In a phase 2 study, fezakinumab demonstrated good efficacy results (expressed as a significant reduction in SCORAD) in the treatment of patients with moderate-to-severe AD, stratified by cutaneous IL-22 levels [214]. Fezakinumab also demonstrated a good safety profile. The most common adverse effect is represented by upper respiratory tract infections.

#### JAK inhibitors

JAK inhibitors act by inhibiting the activity of one or more enzymes of the JAK family (JAK1, JAK2, JAK3), thus interfering with the JAK / STAT signaling pathway. The latter represents one of the main molecular signaling mechanisms for the activity of numerous cytokines and growth factors involved in inflammatory processes and cell replication [207].

Several oral JAK inhibitors such as baricitinib, abrocitinib and upadacitinib are currently being studied for the treatment of patients with AD. First-generation JAK inhibitors (such as baricitinib) target more than one JAK enzyme, while second or newer generation JAK inhibitors (such as upadacitinib and abrocitinib) target specific JAK enzymes [215].

In animal studies, some molecules belonging to the class of JAK inhibitors (such as baricitinib and upadacitinib) have been shown to induce a reduction in fertility and to have teratogenic effects on the fetus [216, 217]. It should be noted that a "black box warning" was issued for upadacitinib and baricitinib, currently in use for rheumatoid arthritis, due to the possible onset of deep vein thrombosis, serious infections and because they are potentially carcinogenic.

#### Baricitinib

Baricitinib, selective for JAK 1 and JAK2 enzymes, is the first orally administered JAK inhibitor [9]. A phase 3 study demonstrated the high efficacy profile of baricitinib in the treatment of moderate-to-severe AD in adult subjects, with a significant reduction in the primary efficacy endpoint (IGA 0/1) at doses of 4 and 2 mg, compared to placebo [218].

In animal studies, a dose of baricitinib 20 times higher than that recommended for the treatment of AD has been shown to induce reduced fertility and teratogenic effects on fetuses [216, 217]. The current recommendation is therefore to avoid the use of baricitnib during pregnancy and to discontinue therapy with baricitinib at least 1 month before conception [216, 217].

#### Upadacitinib

Upadacitinib is a second-generation JAK inhibitor, selective for the JAK1 enzyme. Upadacitinib was developed precisely to improve the safety profile, minimizing the effects related to the inhibition of JAK2 and JAK3 [219]. It has been hypothesized that a higher specificity for the inhibition of JAK1 might reduce the dose-related toxicity of the drug, without leading to a significant reduction in its efficacy [219].

A phase 2b study demonstrated the clinical efficacy of upadacitinib in the treatment of patients with moderate-severe AD, defined by a significant percentage reduction in the EASI score in patients treated with the drug vs. placebo [220].

In animal studies, upadacitinib has been shown to induce fetal teratogenic effects and reduce fetal body weight (EU summary of product characteristics: RINVOQ [221].

The limited data currently available are not sufficient to assess the risk related to drug administration in pregnant women [217]. Pregnant women should be advised that exposure to this drug may induce malformations in the fetus, and effective contraception is recommended in women of childbearing potential during treatment with upadacitinib and for 4 weeks following the last dose of the drug (EU summary of product characteristics: RINVOQ [209–218].

#### Abrocitinib

Abrocitinib is a second-generation JAK inhibitor and is specific for the JAK1 enzyme. A phase 2b study evaluated the efficacy and safety profile of abrocitinib for the treatment of adults with moderate-to-severe AD, reporting a significant clinical improvement of the disease expressed by a reduction in the EASI score and the achievement of an IGA score of 0/1 in patients receiving abrocitinib 200 mg vs. placebo [222].

### Allergen-specific immunotherapy

A percentage of patients with AD may have an increase in total serum IgE and be sensitized to aeroallergens such as house dust mites (HDM) [223].

The efficacy of allergen-specific immunotherapy (AIT) is now well documented both in allergy to Hymenoptera venom and in allergic rhinitis with or without asthma. However, the role of AIT in the therapy of AD is still debated [223].

Some clinical trials have evaluated the clinical efficacy of AIT, administered subcutaneously (SCIT) and sublingually (SLIT), in adults and children with AD. These studies, conducted on small populations, have shown contradictory results regarding actual efficacy [224–227]. More recently, Liu et al. [228] evaluated 239 patients treated with HDM SLIT for 36 weeks demonstrating clinical improvement in subjects with moderate to severe AD. Galli et al. [229] conducted the first controlled study of 60 children with AD and found no significant benefit after 3 years of HDM SLIT. In 2007 Pajno et al. [230] treated 56 patients between 5 and 16 years of age in a double-blind for 18 months, obtaining significant results in patients with mild to moderate AD. Subsequent studies have confirmed the safety and efficacy of SLIT, although conducted on selected populations and with different AIT administration schedules. In recent years, several systematic reviews and meta-analysis studies on both routes of administration, SCIT and SLIT, have come to controversial conclusions and no official recommendations could be made [228].

In 2017, the guidelines of the American Academy of Dermatology [231] confirmed that the available data did not allow to support the use of AIT in AD. However, as in 2018, a Joint Task Force of the European Academy of Dermatology [8] suggested, instead, to consider AIT in selected patients with moderate-severe AD, allergic sensitization to HDM and clinical exacerbation after the exposure to the causative allergen.

It should be noted that to date there are no studies to support the use of AIT in individuals with AD and allergic sensitization to allergens other than HDM, such as dogs and cats. Currently, in the literature, there is only one observational uncontrolled study on a population of 19 adults with AD and sensitization to cat and/or dog dander, in which a marked improvement in skin symptoms, a reduction in specific IgE, and an increase in IgG4 after 2-5 years of SCIT are documented [232]. AIT could be a potential treatment option in patients with severe AD, associated with other allergic comorbidities, and allergy to dogs and cats, if exposure to the allergen cannot be avoided, although further studies are needed to confirm this therapeutic indication. Also, the identification of specific biomarkers capable of predicting the actual clinical efficacy of AIT in different patients is highly needed, as these could represent a decisive element for an adequate selection of the subjects to be treated.

### Thermal therapy

Although the literature data suggest the possible beneficial role of thermal treatments in dermatological diseases, this is nevertheless still debated due to the difficulty of standardizing adequate methods of investigation [233].

Bicarbonate-calcium-magnesium waters are more suitable for the treatment of AD. Retrospective studies have shown beneficial effects of climate treatment and Dead Sea salts in patients with AD, in the absence of major side effects [234]. An open-label, randomized controlled study evaluated the clinical efficacy of the combination of balneotherapy in a 10% Dead Sea salt solution and NBUVB phototherapy compared to phototherapy alone, finding an improvement in AD in patients undergoing balneotherapy [235].

Several studies have reported clinical and quality of life improvement in AD patients after 3 weeks of treatment at the Avène spa (Avène-les-Bains, France) [236–239]. Another study, which involved AD patients treated with 3 weeks of balneotherapy at the La Roche-Posay spa in France, showed a significant improvement in AD in terms of EASI score, quality of life, itching and xerosis [240].

An open-label, randomized controlled study on the efficacy and safety of thermal balneotherapy in children was conducted at the Terme di Comano (Comano Terme, Trento, Italy) on 104 children (1-14 years) with mild-moderate AD. Patients were alternatively assigned to balneotherapy or TCSs for 2 weeks. Four months after the end of treatment, a significant difference in the number and duration of exacerbations was found in favor of the balneotherapy group, with a significant reduction in TCS consumption and days of acute AD [241].

More recently, studies have focused on the microbiological properties of thermal waters and the anti-inflammatory and immunomodulating effects of the new microbial entities discovered in them [242–247]. A new microorganism, called *Aquaphilus dolomiae* (Neisseriaceae), was found in the thermal water of Avène. The *Aquaphilus dolomiae* extract seems to counteract the effect of the cutaneous *S. aureus* secretome isolated from atopic children on the proliferation of CD4 + T cells [243]. The regulatory activity of the ES0 extract of *Aquaphilus dolomiae* on inflammation associated with itching, on alteration of the skin barrier and the innate and adaptive immune response was also described in vitro [244]. These effects suggest the potential role of this extract inserted in topical preparations for the treatment of AD. The thermal water of La Roche-Posay also contains specific non-pathogenic minerals and microbes that can affect the human microbiota. Its microbial composition was characterized by 16S next-generation sequencing metagenomic techniques. The main characteristics are a high bacterial diversity, a low bacterial concentration, and a predominance of Gram-negative bacteria. These bacteria could affect the skin microbiota and therefore the water itself could be considered a probiotic [245]. The effect of thermal balneotherapy on the skin microbiota was evaluated in 31 patients with AD, both on lesional and non-lesional skin sites, at the beginning and end of treatment. Biodiversity of the skin microbiota (measured by Shannon index) improved after 21 days of thermal balneotherapy. Furthermore, a reduction of Firmicutes in particular of *Staphilococci spp* with a significant increase of Gram-negative bacteria was found [246]. The Microbial Genomics Laboratory of the CIBIO Department of the University of Trento (Trento, Italy) has carried out the first characterization study of the groundwater microbiome (Antica Fonte di Comano Terme) with high-resolution sequencing technology (NGS shotgun sequencing). The study found a stable microbiome over time, made up of more than 250 species of which about half had never been described before. The genomes of these bacteria contain thousands of genes and their functions are yet to be characterized. Furthermore, more than 100 bacterial strains have been isolated in pure culture and characterized for various bioactivities [247]. The anti-inflammatory activity found in various strains has proved to be of particular interest for potential future applications in the dermo-cosmetic sector to support the treatment of skin diseases. Although further studies are needed to further clarify these initial data, one bacterial strain with potential immunomodulatory properties has been described as a new bacterial species, the *Mesorhizobium comanense* [247].

In conclusion, emerging evidence seems to support the hypothesis that the therapeutic effects of different thermal waters are due to the concomitance of physical and chemical effects, in conjunction with the immunological and microbiological properties of the waters [247]. Further evidence is needed to explain the role of non-pathogenic bacteria present in thermal waters in promoting skin microbiota diversity and any beneficial effects in AD patients.

### Therapeutic education

The management of skin manifestations and pruritus in children with AD, especially the early-onset phenotype, would require multidisciplinary support for the patient's family, covering bio-pharmacological, educational and psychological/psychotherapeutic aspects. Therapeutic education, as defined by the WHO, also includes a personalized plan to be built in collaboration with the patient and his family.

The anxiety generated by the complex therapeutic management of skin lesions and pruritus and the emotional and economic burden of AD therapies can compromise the quality of life of the entire family and affect compliance with treatment.

An ideal model of integrated management should include the coordinated intervention of a multidisciplinary team composed of the medical specialist (Pediatrician, Allergist, Dermatologist), the psychologist/psychotherapist and the Nurse, and be oriented towards improving the disease and the quality of life of the children and their families [248].

The educational component plays an equally fundamental role within the therapeutic plan [249] (Ministero della Salute-Direzione Generale della Programmazione Sanitaria “Piano Nazionale della Cronicità”. Febbraio 2016)

The role of education is important for any degree of severity of AD since the impact on the quality of life of this disease can be relevant even in non-severe forms [250, 251]. Any educational intervention must be preceded by an unhurried listening to the convictions, difficulties, expectations and prospects of the patient and the family. Indeed, therapeutic education puts the patient and caregivers at the center of the educational process and, in an ideal model, puts in place an integrated and multidisciplinary intervention. The educational approach must be modulated especially towards patients and families with special needs (poverty, social hardship, language difficulties).

Although in many clinical settings such integration of professional figures is a utopian model, this model can still be used as a guide for the specialists, albeit in a simplified form [248]. In general, a synergy must be established at least between the general practitioner/pediatrician and the specialist pediatrician, who recognizes the following as fundamental in the therapeutic approach:

- Throughout information on the disease and related comorbidities
- The reasoned involvement of the patient and those who assist him in therapeutic choices
- Training on the recognition of disease exacerbations
- Training on the use of non-pharmacological treatments (baths, local therapy, bandages)
- The availability of informative and explanatory material (in paper or digital form)
- Short-term monitoring to assess how much of the information and teachings have been withheld and what new questions these teachings have prompted
- Long-term follow-up to evaluate therapeutic outcomes and discuss any new options
- The willingness to educate the school and/or sports environment

The 2018 European Guidelines on the management of AD [7] emphasize that poor adherence to therapies is the most important factor limiting treatment outcomes [252]. Poor adherence to therapeutic indications may be due to various factors, including stress [253] and intra-family psychological and psychodynamic influences, known triggers capable of directly influencing the clinical course of AD and favoring severe exacerbations of eczematous skin lesions [254]. A 2014 Cochrane review assessed ten randomized controlled clinical trials of psychological or educational interventions, in addition to conventional therapy, for AD in children, and reported that educational interventions (such as multi professional eczema interventions and nurse-led clinics) may lead to improvements in AD severity and quality of life [255]. Based on such conclusions, the 2018 European Guidelines concluded that “patient education programs are recommended as an adjunct to conventional therapy of AE” (grade of recommendation 1a, A) [15]. The Guidelines report the following grades of evidence regarding patient educational programs:

- Structured age-related multidisciplinary educational group training programs have the greatest evidence-based benefits (Eczema Schools). (1a)
- Workshops on AD lead to improved severity scores, greater adherence to eczema management, better itch control and additional psychological benefit. (2a, 2b)
- Nurse-managed programs result in more effective use of topical therapies. (3b)
- Nurse-led programs result in improved severity scores. (2a)
- Some evidence suggests that an online model of direct access to follow-up dermatological care is equivalent to the classic follow-up visit for patients with AD. (2a)
- There is no evidence of change in severity scores obtained with self-directed educational programs.

The topic of patient educational programs was also addressed in the Position Paper of 2020 [38] which still recommends participation in a therapeutic education program for AD for all patients with moderate and severe form, underlining how poor adherence to the prescribed treatment is the most frequent condition leading to a therapeutic failure [256]. There is therefore a significant and urgent need for physicians to ensure that their patients are educated and confident in the use of prescribed drugs to gain control of the disease and of itching. The educational program, in group or individual, should be started as early as possible and should consider both the physical and emotional aspects of the patient and family, providing practical guidance for the home management of AD [257]. To improve the quality of life, it is also crucial to teach distraction techniques to control itching, which has a strong negative impact on patients and the entire family [258]. The choice of distraction techniques, best known for pain management, depends on several factors: age, personality, patient preferences, motivation, and emotions of the reference figures.

### Summary BOX

Because AD ​​is a complex disease, both from pathogenetic and clinical standpoints, management is also affected by this complexity. The presence of different clinical phenotypes of AD implies the need for an individualized and multidisciplinary approach in which the interaction between the primary care pediatrician, the pediatric allergist, and the pediatric dermatologist is crucial to finding the best management strategy. In the manuscript, the latest shreds of evidence on pharmacological and non-pharmacological therapies for AD are reviewed. This narrative review aims to define a pathway to appropriately managing children and adolescents with moderate-severe atopic dermatitis (AD). A flow chart for the possible multidisciplinary management of pediatric moderate-severe AD in the Italian Health Care System is also reported (Table 10).

**Table 10:** Flow chart for the possible multidisciplinary management of pediatric moderate-severe atopic dermatitis in italy

	Healthcare facility	Actor	Action
Phase 1	General Practice	Primary Care Pediatrician and General Practitioner	• Management of mild AD, • Promotion of basic management strategies of AD (i.e. moisturizing, mild-moderate potency topical steroids, antibiotics) • Refer moderate-severe AD patients to specialists
Phase 2	Community or 1-2^nd^ levels Hospitals	Pediatric Allergist and/or Dermatologist	• Management of mild to moderate AD (SCORAD <50) using I-II line therapy (eg. Wet-wrap therapy; phototherapy) • Specific testing, if needed (eg. skin prick test and/or serum specific IgE testing, patch test, biopsy) • Educational therapy • To refer the patient to a University or 3^rd^ level Hospital if severe AD (SCORAD >50) or difficult to treat AD patient
Phase 3	University or 3^rd^ level Hospitals	Pediatric allergist AND dermatologist	• Re-evaluation of differential diagnosis and comorbidities • Definition of the baseline severity (SCORAD and EASI) and further specific testing, if needed • To start III line therapies for severe AD (eg. add on immunosuppressants, biologics) and management of eventual comorbidities • Involvement of other specialist health care professionals (eg. immunologist, psychologist, dietician)

## Abbreviations

AD: Atopic dermatitis
CsA: Cyclosporin A
EMA: European Medicines Agency
FDA: Food and Drug Administration
GM-CSF: Granulocyte-macrophage colony-stimulating factor
IL: Interleukin
INF-γ: Interferon-gamma
MRSA: Methicillin-resistant Staphylococcus aureus
NaOCl: Sodium hypochlorite
NFκB: Nuclear Factor kappa-light-chain-enhancer of activated B cells
tTAC: topical tacrolimus
TCIs: Topical calcineurin inhibitors
TCSs: Topical corticosteroids
tPIM: Topical pimecrolimus

## References

[1] Thomas KS, Apfelbacher CA, Chalmers JR, Simpson E, Spuls PI, Gerbens LAA, et al. Recommended core outcome instruments for health-related quality of life, long-term control and itch intensity in atopic eczema trials: results of the HOME VII consensus meeting. Br J Dermatol. 2020. 10.1111/bjd.19673. Epub ahead of print. 10.1111/bjd.19673 33179283

[2] Severity scoring of atopic dermatitis: the SCORAD index (1993). Consensus report of the European task force on atopic dermatitis. Dermatology.

[3] Hanifin JM, Thurston M, Omoto M, EASI Evaluator Group (2001). The eczema area and severity index (EASI): assessment of reliability in atopic dermatitis. Exp Dermatol.

[4] Conic RZ, Tamashunas NL, Damiani G, Fabbrocini G, Cantelli M, Young Dermatologists Italian Network, & Bergfeld, W. F. (2020) (2020). Comorbidities in pediatric alopecia areata. J Eur Acad Dermatol Venereol.

[5] Perugia C, Saraceno R, Ventura A (2017). Atopic dermatitis and dental manifestations. G Ital Dermatol Venereol.

[6] Damiani G, Gironi LC, Pacifico A, Pigatto P, Malagoli P, Bindi M (2021). Cutaneous and oral comorbidities in patients with geographic tongue: a multicenter multidisciplinary cross-sectional observational study. J Biol Regul Homeost Agents.

[7] Damiani G, Calzavara-Pinton P, Stingeni L, Hansel K, Cusano F, “Skin Allergy” Group of SIDeMaST, “ADOI” (Associazione Dermatologi Ospedalieri Italiani), “SIDAPA” (Società Italiana di Dermatologia Allergologica, Professionale e Ambientale), & Pigatto P (2019). Italian guidelines for therapy of atopic dermatitis-Adapted from consensus-based European guidelines for treatment of atopic eczema (atopic dermatitis). Dermatologic Therap.

[8] Wollenberg A, Barbarot S, Bieber T, Christen-Zaech S, Deleuran M, Fink-Wagner A (2018). Consensus-based European guidelines for treatment of atopic eczema (atopic dermatitis) in adults and children: part I. J Eur Acad Dermatol Venereol.

[9] Katoh N, Ohya Y, Ikeda M, Ebihara T, Katayama I, Saeki H (2019). Clinical practice guidelines for the management of atopic dermatitis 2018. J Dermatol.

[10] Chiricozzi A, Comberiati P, D'Auria E, Zuccotti G, Peroni DG (2020). Topical corticosteroids for pediatric atopic dermatitis: Thoughtful tips for practice. Pharmacol Res.

[11] Spergel JM (2008). Immunology and treatment of atopic dermatitis. Am J Clin Dermatol.

[12] Norris DA (2005). Mechanisms of action of topical therapies and the rationale for combination therapy. J Am Acad Dermatol.

[13] Sigurgeirsson B, Boznanski A, Todd G, Vertruyen A, Schuttelaar ML, Zhu X (2015). Safety and efficacy of pimecrolimus in atopic dermatitis: a 5-year randomized trial. Pediatrics.

[14] Chiricozzi A, Belloni Fortina A, Galli E, Girolomoni G, Neri I, Ricci G (2019). Current therapeutic paradigm in pediatric atopic dermatitis: Practical guidance from a national expert panel. Allergol Immunopathol (Madr).

[15] Wollenberg A, Barbarot S, Bieber T, Christen-Zaech S, Deleuran M, Fink-Wagner A (2018). Consensus-based European guidelines for treatment of atopic eczema (atopic dermatitis) in adults and children: part II. J Eur Acad Dermatol Venereol.

[16] Hoeger PH, Kinsler V, Yan AC, Harper J, Oranje AP, Bodemer CB, Larralde M, Luk D, Mendiratta V, Purvis D. Harper's Textbook of Pediatric Dermatology, 2 Volume Set, 4th Edition. 2019.

[17] Danby SG, Draelos ZD, Gold LFS, Danby SG, Draelos ZD, Gold LFS (2020). Vehicles for atopic dermatitis therapies: more than just a placebo. J Dermatolog Treat.

[18] Long CC, Finlay AY (1991). The finger-tip unit–a new practical measure. Clin Exp Dermatol.

[19] Hong E, Smith S, Fischer G (2011). Evaluation of the atrophogenic potential of topical corticosteroids in pediatric dermatology patients. Pediatr. Dermatol.

[20] Thomas KS, Armstrong S, Avery A, Po AL, O'Neill C, Young S (2002). Randomised controlled trial of short bursts of a potent topical corticosteroid versus prolonged use of a mild preparation for children with mild or moderate atopic eczema. BMJ.

[21] Brazzini B, Pimpinelli N (2002). New and established topical corticosteroids in dermatology: clinical pharmacology and therapeutic use. Am J Clin Dermatol.

[22] Wood Heickman LK, Davallow Ghajar L, Conaway M, Rogol AD (2018). Evaluation of hypothalamic-pituitary-adrenal axis suppression following cutaneous use of topical corticosteroids in children: a meta-analysis. Horm Res Paediatr.

[23] Friedlander SF, Hebert AA, Allen DB, Fluticasone Pediatrics Safety Study Group (2002). Safety of fluticasone propionate cream 0.05% for the treatment of severe and extensive atopic dermatitis in children as young as 3 months. J Am Acad Dermatol.

[24] Blume-Peytavi U, Wahn U (2011). Optimizing the treatment of atopic dermatitis in children: a review of the benefit/risk ratio of methylprednisolone aceponate. J Eur Acad Dermatol Venereol.

[25] Berth-Jones J, Damstra RJ, Golsch S, Livden JK, Van Hooteghem O, Allegra F (2003). Twice weekly fluticasone propionate added to emollient maintenance treatment to reduce risk of relapse in atopic dermatitis: randomised, double blind, parallel group study. BMJ.

[26] van Velsen SG, Knol MJ, van Eijk RL, de Vroede MA, de Wit TC, Lam MG (2010). Bone mineral density in children with moderate to severe atopic dermatitis. J Am Acad Dermatol.

[27] Zhao M, Liang Y, Shen C, Wang Y, Ma L, Ma X (2020). Patient education programs in pediatric atopic dermatitis: a systematic review of randomized controlled trials and meta-analysis. Dermatol Ther (Heidelb).

[28] Li AW, Yin ES, Antaya RJ (2017). Topical corticosteroid phobia in atopic dermatitis: a systematic review. JAMA Dermatol.

[29] Moret L, Anthoine E, Aubert-Wastiaux H, Le Rhun A, Leux C, Mazereeuw-Hautier J (2013). TOPICOP©: a new scale evaluating topical corticosteroid phobia among atopic dermatitis outpatients and their parents. PLoS One.

[30] Patrizi A, Gurioli C. Corticosteroidi topici in Dermatologia: una review. ConferenceInsight2014[http://www.springerhealthcare.it/_upload/oaj/20140630140434. pdf].

[31] Carr WW (2013). Topical calcineurin inhibitors for atopic dermatitis: review and treatment recommendations. Paediatr Drugs.

[32] Chehade A, Rao J, Topical Calcineurin Inhibitors (2021). Topical Calcineurin Inhibitors Comprehensive Dermatologic Drug Therapy.

[33] Nakahara T, Morimoto H, Murakami N, Furue M (2018). Mechanistic insights into topical tacrolimus for the treatment of atopic dermatitis. Pediatr Allergy Immunol.

[34] Pereira U, Boulais N, Lebonvallet N, Pennec JP, Dorange G, Misery L (2010). Mechanisms of the sensory effects of tacrolimus on the skin. Br J Dermatol.

[35] Mandelin JM, Remitz A, Virtanen HM, Malmberg LP, Haahtela T, Reitamo S (2010). A 10-year open follow-up of eczema and respiratory symptoms in patients with atopic dermatitis treated with topical tacrolimus for the first 4 years. J Dermatolog Treat.

[36] Jovanović M, Golušin Z (2016). Nonsteroidal Topical Immunomodulators in Allergology and Dermatology. Biomed Res Int.

[37] Billich A, Aschauer H, Aszódi A, Stuetz A (2004). Percutaneous absorption of drugs used in atopic eczema: pimecrolimus permeates less through skin than corticosteroids and tacrolimus. Int J Pharmaceutics.

[38] Chittock J, Brown K, Cork MJ (2015). Comparing the effect of a twice-weekly tacrolimus and betamethasone valerate dose on the subclinical epidermal barrier defect in atopic dermatitis. Acta Derm Venereol.

[39] Dähnhardt D, Bastian M, Dähnhardt-Pfeiffer S, Buchner M, Fölster-Holst R (2020). Comparing the effects of proactive treatment with tacrolimus ointment and mometasone furoate on the epidermal barrier structure and ceramide levels of patients with atopic dermatitis. J Dermatolog Treat.

[40] Remitz A, De Pità O, Mota A, Serra-Baldrich E, Vakirlis E, Kapp A (2018). Position statement: topical calcineurin inhibitors in atopic dermatitis. Eur Acad Dermatol.

[41] Chen SL, Yan J, Wang FX (2010). Two topical calcineurin inhibitors for the treatment of atopic dermatitis in pediatric patients: a meta-analysis of randomized clinical trials. J Dermatol Treat.

[42] El-Batawy MM, Bosseila MA, Mashaly HM, Hafez VS (2009). Topical calcineurin inhibitors in atopic dermatitis: a systematic review and meta-analysis. J Dermatol Sci.

[43] Ohtsuki M, Morimoto H, Nakagawa H (2018). Tacrolimus ointment for the treatment of adult and pediatric atopic dermatitis: review on safety and benefits. J Dermatol.

[44] Siegfried EC, Jaworski JC, Kaiser JD, Hebert AA (2016). Systematic review of published trials: long-term safety of topical corticosteroids and topical calcineurin inhibitors in pediatric patients with atopic dermatitis. BMC Pediatr.

[45] Wollenberg A, Christen-Zäch S, Taieb A, Paul C, Thyssen JP, de Bruin-Weller M (2020). ETFAD/EADV Eczema task force 2020 position paper on diagnosis and treatment of atopic dermatitis in adults and children. J Eur Acad Dermatol Venereol.

[46] Luger T, Augustin M, Lambert J, Paul C, Pincelli C, Torrelo A (2021). Unmet medical needs in the treatment of atopic dermatitis in infants: an expert consensus on safety and efficacy of pimecrolimus. Pediatr Allergy Immunol.

[47] Sher LG, Chang J, Patel IB, Balkrishnan R, Fleischer AB (2012). Relieving the pruritus of atopic dermatitis: a meta-analysis. Acta Derm Venereol.

[48] Thaci D, Chambers C, Sidhu M, Dorsch B, Ehlken B, Fuchs S (2010). Twice weekly treatment with tacrolimus 0.03% ointment in children with atopic dermatitis: clinical efficacy and economic impact over 12 months. J Eur Acad Dermatol Venereol.

[49] Katoh N, Ohya Y, Ikeda M, Ebihara T, Katayama I, Saeki H (2020). Japanese guidelines for atopic dermatitis 2020. Allergol Int.

[50] Wongpiyabovorn J, Soonthornchai W, Wilantho A, Palasuk M, Payungporn S, Sodsai P (2019). Effect of tacrolimus on skin microbiome in atopic dermatitis. Allergy.

[51] Beck KM, Seitzman GD, Yang EJ, Sanchez IM, Liao W, Ocular Co-Morbidities of Atopic Dermatitis (2019). Part II: ocular disease secondary to treatments. Am J Clin Dermatol..

[52] Ollech A, Yousif R, Kruse L, Wagner A, Kenner-Bell B, Chamlin S (2020). Topical calcineurin inhibitors for pediatric periorificial dermatitis. J Am Acad Dermatol.

[53] Radovic TC, Kostovic K, Ceovic R, Mokos ZB (2017). Topical calcineurin inhibitors and malignancy risk. Int J Cancer Manag.

[54] Castellsague J, Kuiper JG, Pottegård A, Anveden Berglind I, Dedman D, Gutierrez L (2018). A cohort study on the risk of lymphoma and skin cancer in users of topical tacrolimus, pimecrolimus, and corticosteroids (Joint European Longitudinal Lymphoma and Skin Cancer Evaluation - JOELLE study). Clin Epidemiol.

[55] Margolis DJ, Abuabara K, Hoffstad OJ, Wan J, Raimondo D, Bilker WB (2015). Association between malignancy and topical use of pimecrolimus. JAMA Dermatol.

[56] Paller AS, Fölster-Holst R, Chen SC, Diepgen TL, Elmets C, Margolis DJ (2020). No evidence of increased cancer incidence in children using topical tacrolimus for atopic dermatitis. J Am Acad Dermatol.

[57] Rafiq M, Hayward A, Warren-Gash C, Denaxas S, Gonzalez-Izquierdo A, Lyratzopoulos G (2020). Allergic disease, corticosteroid use, and risk of hodgkin lymphoma: a United Kingdom nationwide case-control study. J Allergy Clin Immunol.

[58] Asgari MM, Tsai AL, Avalos L, Sokil M, Quesenberry CP (2020). Association between topical calcineurin inhibitor use and keratinocyte carcinoma risk among adults with atopic dermatitis. JAMA Dermatol.

[59] Wang V, Boguniewicz J, Boguniewicz M, Ong PY (2021). The infectious complications of atopic dermatitis. Ann Allergy Asthma Immunol.

[60] Allen HB, Vaze ND, Choi C, Hailu T, Tulbert BH, Cusack CA (2014). The presence and impact of biofilm- producing staphylococci in atopic dermatitis. JAMA Dermatol.

[61] Williams MR, Gallo RL (2017). Evidence that human skin microbiome dysbiosis promotes atopic dermatitis. J Invest Dermatol.

[62] Kong HH, Segre JA (2012). Skin microbiome: looking back to move forward. J Invest Dermatol.

[63] Kong HH, Oh J, Deming C, Conlan S, Grice EA, Beatson MA (2012). Temporal shifts in the skin microbiome associated with disease flares and treatment in children with atopic dermatitis. Genome Res.

[64] Langan SM, Irvine AD, Weidinger S (2020). Atopic dermatitis. Lancet.

[65] Petry V, Bessa GR, Poziomczyck CS, Oliveira CF, Weber MB, Bonamigo RR (2012). Bacterial skin colonization and infections in patients with atopic dermatitis. An Bras Dermatol.

[66] Kim J, Kim BE, Ahn K, Leung DYM (2019). Interactions between atopic dermatitis and staphylococcus aureus infection: clinical implications. Allergy Asthma Immunol Res.

[67] Stephanie M (2018). Bacterial colonization, overgrowth, and superinfection in atopic dermatitis. Clin Dermatol.

[68] Rosen T, Albareda N, Rosenberg N, Alonso FG, Roth S, Zsolt I (2018). Efficacy and safety of ozenoxacin cream for treatment of adult and pediatric patients with impetigo a randomized clinical trial. Jama Dermatol.

[69] Bonamonte D, Belloni Fortina A, Neri L (2014). Fusidic acid in skin infections and infected atopic eczema. G Ital Dermatol Venereol.

[70] Bonamonte D, Belloni Fortina A, Neri L, Patrizi A (2020). Topical antibiotics in the dermatological clinical practice: Indications, efficacy, and adverse effects. Dermatol Ther.

[71] Rangel SM, Paller AS (2018). Bacterial colonization, overgrowth, and superinfection in atopic dermatitis. Clin Dermatol.

[72] Chiu LS, Chow VC, Ling JM, Hon KL (2010). Staphylococcus aureus carriage in the anterior nares of close contacts of patients with atopic dermatitis. Arch Dermatol.

[73] Clebak KT, Malone MA (2018). Skin infections. Prim Care.

[74] George SM, Karanovic S, Harrison DA, Rani A, Birnie AJ, Bath-Hextall FJ (2019). Interventions to reduce staphylococcus aureus in the management of eczema. Cochrane Database Syst Rev.

[75] Stevens DL, Bisno AL, Chambers HF, Dellinger EP, Goldstein EJ, Gorbach SL (2014). Practice guidelines for the diagnosis and management of skin and soft tissue infections: 2014 update by the infectious diseases society of America. Clin Infect Dis.

[76] Montravers P, Snauwaert A, Welsch C (2016). Current guidelines and recommendations for the management of skin and soft tissue infections. Curr Opin Infect Dis.

[77] Koch L, Cerpes U, Binder B, Cerroni L (2021). Disseminated bullous impetigo in atopic dermatitis ('eczema staphylococcatum'). Eur Acad Dermatol Venereol.

[78] Galli L, Venturini E, Bassi A, Gattinara GC, Chiappini E, Defilippi C, Italian Pediatric Infectious Diseases Society; Italian Pediatric Dermatology Society (2019). Common community-acquired bacterial skin and soft-tissue infections in children: an intersociety consensus on impetigo, abscess, and cellulitis treatment. Clin Ther.

[79] Harkins CP, Holden MTG, Irvine AD (2019). Antimicrobial resistance in atopic dermatitis: need for an urgent rethink. Ann Allergy Asthma Immunol.

[80] Majewski S, Bhattacharya T, Asztalos M, Bohaty B, Durham KC, West DP (2019). Sodium hypochlorite body wash in the management of Staphylococcus aureus–colonized moderate-to-Severe atopic dermatitis in infants, children, and adolescents. Pediatric Dermatol.

[81] Leung TH, Zhang LF, Wang J, Ning S, Knox SJ, Kim SK (2013). Topical hypochlorite ameliorates NF-kappaB-mediated skin diseases in mice. J Clin Invest.

[82] Maarouf M, Shi VY (2018). Bleach for atopic dermatitis. Dermatitis.

[83] Eriksson S (2017). Antibacterial and antibiofilm effects of sodium hypochlorite against staphylococcus aureus isolates derived from patients with atopic dermatitis. Br J Dermatol.

[84] Rerinck HC, Kamann S, Wollenberg A (2006). Eczema herpeticatum: pathogenese und therapie [Eczema herpeticum: Pathogenesis and therapy]. Hautarzt.

[85] Seegräber M, Worm M, Werfel T, Svensson A, Novak N, Simon D (2020). Recurrent eczema herpeticum - a retrospective European multicenter study evaluating the clinical characteristics of eczema herpeticum cases in atopic dermatitis patients. J Eur Acad Dermatol Venereol.

[86] Leung DY (2013). Why is eczema herpeticum unexpectedly rare?. Antiviral Res.

[87] Ong PY, Leung DYM (2016). Bacterial and viral infections in atopic dermatitis: a comprehensive review. Clin Rev Allergy Immunol.

[88] Guzman AK, Schairer DO, Garelik JL, Cohen SR (2018). Safety and efficacy of topical cantharidin for the treatment of pediatric molluscumcontagiosum: a prospective, randomized, double-blind, placebo-controlled pilot trial. Int J Dermatol.

[89] Berger EM, Orlow SJ, Patel RR, Schaffer JV (2012). Experience with molluscum contagiosum and associated inflammatory reactions in a pediatric dermatology practice: the bump that rashes. Arch Dermatol.

[90] Mathes EF, Oza V, Frieden IJ, Cordoro KM, Yagi S, Howard R (2013). "Eczema coxsackium" and unusualcutaneousfindings in an enterovirusoutbreak. Pediatrics.

[91] Neri I, Dondi A, Wollenberg A, Ricci L, Ricci G, Piccirilli G (2016). Atypical forms of hand, foot, and mouth disease: a prospective study of 47 Italian children. Pediatr Dermatol.

[92] Johnson VK, Hayman JL, McCarthy CA, Cardona ID (2014). Successful treatment of eczema coxsackium with wet wrap therapy and low-dose topical corticosteroid. J Allergy Clin Immunol Pract.

[93] Ikezawa Z, Kondo M, Okajima M, Nishimura Y, Kono M (2004). Clinical usefulness of oral itraconazole, an antimycotic drug, for refractory atopic dermatitis. Eur J Dermatol.

[94] Darabi K, Hostetler SG, Bechtel MA, Zirwas M (2009). The role of Malassezia in atopic dermatitis affecting the head and neck of adults. J Am Acad Dermatol..

[95] Saunte DML, Gaitanis G, Hay RJ (2020). Malassezia-associated skin diseases, the use of diagnostics and treatment. Front Cell Infect Microbiol..

[96] Guglielmo A, Sechi A, Patrizi A, Gurioli C, Neri I (2021). Head and neck dermatitis, a subtype of atopic dermatitis induced by Malassezia spp: clinical aspects and treatment outcomes in adolescent and adult patients. Pediatr Dermatol.

[97] Mayser P, Kupfer J, Nemetz D, Schäfer U, Nilles M, Hort W (2006). Treatment of head and neck dermatitis with ciclopiroxolamine cream - results of a double-blind, placebo- controlled study. Skin Pharmacol Physiol.

[98] Morita E, Hide M, Yoneya Y, Kannbe M, Tanaka A, Yamamoto S (1999). An assessment of the role of Candida albicans antigen in atopic dermatitis. J Dermatol.

[99] Javad G, Taheri Sarvtin M, Hedayati MT, Hajheydari Z, Yazdani J (2015). Evaluation of Candida colonization and specific humoral responses against Candida albicans in patients with atopic dermatitis. Biomed Res Int.

[100] Thammahong A, Kiatsurayanon C, Edwards SW, Rerknimitr P, Chiewchengchol D (2020). The clinical significance of fungi in atopic dermatitis. Int J Dermatol.

[101] Soeberdt M, Kilic A, Abels C (2020). Small molecule drugs for the treatment of pruritus in patients with atopic dermatitis. Eur J Pharmacol.

[102] Fahrbach K, Tarpey J, Washington EB, Hughes R, Thom H, Neary MP (2020). Crisaborole ointment, 2%, for treatment of patients with mild-to-moderate atopic dermatitis: systematic literature review and network meta-analysis JC. Dermatol Ther (Heidelb).

[103] Paller AS, Tom WL, Lebwohl MG, Paller AS, Tom WL, Lebwohl MG (2016). Efficacy and safety of crisaborole ointment, a novel, nonsteroidal phosphodiesterase 4 (PDE4) inhibitor for the topical treatment of atopic dermatitis (AD) in children and adults. J Am Acad Dermatol.

[104] Solimani F, Meier K, Ghoreschi K (2019). Emergin topical and sistemi jak inhibitors in dermatology. Front Immunol.

[105] Rodrigues MA, Torres T (2020). Jak/STA inhibitors for the treatment of atopic dermatitis. J Dermatolog Treat.

[106] Guttman-Yassky E, Hanifin JM, Boguniewicz M, Wollenberg A, Bissonnette R, Purohit V (2019). The role of phosphodiesterase 4 in the pathophysiology of atopic dermatitis and the perspective for its inhibition. Exp Dermatol..

[107] Delgocitinib Dhillon S (2020). First approval. Drugs.

[108] Brar KK, Nicol NH, Boguniewicz M (2019). Strategies for successful management of severe atopic dermatitis. J Allergy Clin Immunol Pract.

[109] Janmohamed SR, Eichenfield LF, Ring J, Gutermuth J (2020). Medical algorithm: diagnosis of atopic dermatitis in early childhood (part II). Allergy.

[110] Cadmus SD, Sebastian KR, Warren D, Hovinga CA, Croce EA, Reveles LA (2019). Efficacy and patient opinion of wet-wrap dressings using 0.1% triamcinolone acetonide ointment vs cream in the treatment of pediatric atopic dermatitis: a randomized split-body control study. Pediatr Dermatol.

[111] Mirza SA (2020). Serum triamcinolone levels during intensive, inpatient wet-dressing therapy. Clin Exp Dermatol.

[112] Witte M, Krause L, Zillikens D, Shimanovich I (2019). Black tea dressings - a rapidly effective treatment for facial dermatitis. J Dermatolog Treat.

[113] Huiling H, Koh MJ, Lee HY, Ang SB (2020). Pilot study of a customized nanotextile wet garment treatment on moderate and severe atopic dermatitis: a randomized clinical trial. Pediatr Dermatol.

[114] Naylor GRS (2009). Fabric-evoked prickle in worsted spun single jersey fabrics part 4: extension from wool to OptimTMfine Fiber. Text Res J.

[115] Gauger A, Mempel M, Schekatz A, Schäfer T, Ring J, Abeck D (2003). Silver-coated textiles reduce Staphylococcus aureus colonization in patients with atopic eczema. Dermatology.

[116] Gauger A, Fischer S, Mempel M, Schaefer T, Foelster-Holst R, Abeck D (2006). Efficacy and functionality of silver-coated textiles in patients with atopic eczema. J Eur Acad Dermatol Venereol.

[117] Zhai H, Dika E, Goldovsky M, Maibach HI (2007). Tape-stripping method in man: comparison of evaporimetric methods. Skin Res Technol.

[118] Hipler UC, Elsner P, Fluhr JW (2006). A new silver-loaded cellulosic fiber with antifungal and antibacterial properties. Curr Probl Dermatol.

[119] Park KY, Jang WS, Yang GW, Rho YH, Kim BJ, Mun SK (2012). A pilot study of silver-loaded cellulose fabric with incorporated seaweed for the treatment of atopic dermatitis. Clin Exp Dermatol.

[120] Araújo CP, Gomes J, Vieira AP, Ventura F, Fernandes JC, Brito C (2013). A proposal for the use of new silver-seaweed-cotton fibers in the treatment of atopic dermatitis. Cutan Ocul Toxicol..

[121] Pluut OA, Bianco C, Jakasa I, Visser MJ, Krystek P, Larese-Filon F (2015). Percutaneous penetration of silver from a silver containing garment in healthy volunteers and patients with atopic dermatitis. Toxicol Lett.

[122] Lopes C, Soares J, Tavaria F, Duarte A, Correia O, Sokhatska O (2015). Chitosan coated textiles may improve atopic dermatitis severity by modulating skin staphylococcal profile: a randomized controlled trial. PLoS One.

[123] Ricci G, Patrizi A, Bendandi B, Menna G, Varotti E, Masi M (2004). Clinical effectiveness of a silk fabric in the treatment of atopic dermatitis. Br J Dermatol.

[124] Ricci G, Patrizi A, Mandrioli P, Specchia F, Medri M, Menna G (2006). Evaluation of the antibacterial activity of a specialsilk textile in the treatment of atopic dermatitis. Dermatology.

[125] Koller DY, Halmerbauer G, Bock A, Engstler G (2007). Action of a silk fabric treated with AEGIS in children with atopic dermatitis: a 3-month trial. Pediatr Allergy Immunol.

[126] Stinco G, Piccirillo F, Valent F (2008). A randomized double-blind study to investigate the clinical efficacy of adding a non-migrating antimicrobial to a special silk fabric in the treatment of atopic dermatitis. Dermatology.

[127] Fontanini C, Berti I, Monasta L, Longo G (2013). DermaSilk in long-term control of infantile atopic dermatitis: a double blind randomized controlled trial. G Ital Dermatol Venereol.

[128] Thomas KS, Bradshaw LE, Sach TH, Cowdell F, Batchelor JM, Lawton S (2017). Randomised controlled trial of silk therapeutic garments for the management of atopic eczema in children: theCLOTHES trial. Health Technol Assess.

[129] Srour J, Wollenberg A (2018). Evaluation of antimicrobial textiles for atopic dermatitis. Br J Dermatol.

[130] Jaros J, Wilson C, Shi VY (2020). Fabric selection in atopic dermatitis: an evidence based review. Am J Clin Dermatol.

[131] Drucker AM, Eyerich K, de Bruin-Weller MS, Thyssen JP, Spuls PI, Irvine AD (2018). Use of systemic corticosteroids for atopic dermatitis: international Eczema council consensus statement. Br J Dermatol.

[132] Yu SH, Drucker AM, Lebwohl M, Silverberg JI (2018). A systematic review of the safety and efficacy of systemic corticosteroids in atopic dermatitis. J Am Acad Dermatol.

[133] Misery L, Belloni Fortina A, El Hachem M, Chernyshov P, von Kobyletzki L, Heratizadeh A (2021). A position paper on the management of itch and pain in atopic dermatitis from the International Society of Atopic Dermatitis (ISAD)/Oriented Patient-Education Network in Dermatology (OPENED) task force. J Eur Acad Dermatol Venereol.

[134] Sidbury R, Davis DM, Cohen DE, Cordoro KM, Berger TG, Bergman JN, American Academy of Dermatology (2014). Guidelines of care for the management of atopic dermatitis: section 3. Management and treatment with phototherapy and systemic agents. J Am Acad Dermatol.

[135] Chan TC, Wu NL, Wong LS (2020). Taiwanese Dermatological Association consensus for the management of atopic dermatitis: A 2020 update. J Formos Med Assoc.

[136] Amor KT, Ryan C, Menter A (2010). The use of cyclosporine in dermatology: part I. J Am Acad Dermatol.

[137] Amber T, Tabassum S (2020). Cyclosporin in dermatology: a practical compendium. Dermatol Ther.

[138] Caproni M, Salvatore E, Cardinali C, Brazzini B, Fabbri P (2000). Soluble CD30 andcyclosporin in severe atopic dermatitis. Int Arch Allergy Immunol.

[139] Roekevisch E, Spuls PI, Kuester D, Limpens J, Schmitt J (2014). Efficacy and safety of systemic treatments for moderate-to-severe atopic dermatitis: a systematic review. J Allergy Clin Immunol.

[140] Schmitt J, Schmitt N, Meurer M (2007). Cyclosporin in the treatment of patients with atopic eczema – a systematic review and meta-analysis. J Eur Acad Dermatol Venereol.

[141] Berth-Jones J, Finlay AY, Zaki I, Tan B, Goodyear H, Lewis-Jones S (1996). Cyclosporine in severe childhood atopic dermatitis: a multicenter study. J Am Acad Dermatol..

[142] Zaki I, Emerson R, Allen BR (1996). Treatment of severe atopic dermatitis in childhood with cyclosporine. Br J Dermatol.

[143] Harper JI, Ahmed I, Barclay G, Lacour M, Hoeger P, Cork MJ (2000). Cyclosporin for severe childhood atopic dermatitis: short course versus continuous therapy. Br J Dermatol.

[144] El-Khalawany MA, Hassan H, Shaaban D, Ghonaim N, Eassa B (2013). Methotrexate versus cyclosporine in the treatment of severe atopic dermatitis in children: a multicenter experience from Egypt. Eur J Pediatr.

[145] Irvine AD, Jones AP, Beattie P, Baron S, Browne F, Ashoor F (2018). A randomized controlled trial protocol assessing the effectiveness, safety and cost-effectiveness of methotrexate vs. ciclosporin in the treatment of severe atopic eczema in children: the TREatment of severe Atopic eczema Trial (TREAT). Br J Dermatol.

[146] Damiani G, Calzavara-Pinton P, Stingeni L, Hansel K, Cusano F, “Skin Allergy” Group of SIDeMaST; “ADOI” (Associazione Dermatologi Ospedalieri Italiani); “SIDAPA” (Società Italiana di Dermatologia Allergologica, Professionale e Ambientale), Pigatto PDM (2019). Italian guidelines for therapy of atopic dermatitis-Adapted from consensus-based European guidelines for treatment of atopic eczema (atopic dermatitis). Dermatol Ther.

[147] Lansang P, Lara-Corrales I, Bergman JN, Hong CH, Joseph M, Kim VHD (2019). Approach to the assessment and management of pediatric patients with atopic dermatitis: a consensus document. Section IV: consensus statements on the assessment and management of pediatric atopic dermatitis. J Cutan Med Surg.

[148] Hernández-Martín A, Noguera-Morel L, Bernardino-Cuesta B, Torrelo A, Pérez-Martin MA, Aparicio-López C (2017). Cyclosporine A for severe atopic dermatitis in children efficacy and safety in a retrospective study of 63 patients. J Eur Acad Dermatol Venereol.

[149] Proudfoot LE, Powell AM, Ayis S, Barbarot S, Baselga Torres E, Deleuran M (2013). The European treatment of severe atopic eczema in children taskforce (TREAT) survey. J Eur Acad Dermatol Venereol.

[150] Drucker AM, Ellis AG, Bohdanowicz M, Mashayekhi S, Yiu ZZN, Rochwerg B (2020). Systemic immunomodulatory treatments for patients with atopic dermatitis: a systematic review and network meta-analysis. JAMA Dermatol.

[151] Cronstein BN, Aune TM (2020). Methotrexate and its mechanisms of action in inflammatory arthritis. Nat Rev Rheumatol.

[152] Weathrhead SC, Wahie S, Reynolds NY (2007). An open label, dose-ranging study of methotrexate for moderate-to-severe adult atopic eczema. Br J Dermatol.

[153] Schram ME, Roekevisch E, Leeflang MM, Bos JD, Schmitt J, Spuls PI (2011). A randomized trial of methotrexate versus azathioprine for severe atopic eczema. J Allergy Clin Immunol.

[154] Dvorakova V, O'Regan GM, Irvine AD (2017). Methotrexate for severe childhood atopic dermatitis: clinical experience in a tertiary center. Pediatr Dermatol.

[155] Anderson K, Putterman E, Rogers RS, Patel D, Treat JR, Castelo-Soccio L (2019). Treatment of severe pediatric atopic dermatitis with methotrexate: a retrospective review. Pediatr Dermatol.

[156] Chavez-Alvarez S, Herz-Ruelas M, Villarreal-Martinez A (2020). Azathioprine: its uses in dermatology. An Bras Dermatol.

[157] Murphy LA, Atherton DJ (2003). Azathioprine as a treatment for severe atopic eczema in children with a partial thiopurine methyl transferase (TPMT) deficiency. Pediatr Dermatol.

[158] Caufield M, Tom WL (2013). Oral azathioprine for recalcitrant pediatric atopic dermatitis: clinical response and thiopurine monitoring. J Am Acad Dermatol.

[159] Chong JH, Koh MJA (2017). Non-topical management of recalcitrant paediatric atopic dermatitis. Arch Dis Child.

[160] Waxweiler WT, Agans R, Morrell DS (2011). Systemic treatment of pediatric atopic dermatitis with azathioprine and mycophenolate mofetil. Pediatr Dermatol.

[161] Heller M, Shin HT, Orlow SJ, Schaffer JV (2007). Mycophenolatemofetil for severe childhood atopic dermatitis: experience in14 patients. Br J Dermatol.

[162] Neuber K, Schwartz I, Itschert G, Dieck AT (2000). Treatment of atopic eczema with oral mycophenolate mofetil. Br J Dermatol.

[163] Scottish Intercollegiate Guidelines Network (SIGN). Management of atopic eczema in primary care. Edinburgh: SIGN; 2011. (SIGN publication no. 125). [March 2011]. Last Accessed February 2021. Available from URL http://www.sign.ac.uk/pdf/sign125.pdf

[164] Silverberg NB, Durán-McKinster C (2017). Special considerations for therapy of pediatric atopic dermatitis. Dermatol Clin.

[165] Lyons JJ, Milner JD, Stone KD (2015). Atopic dermatitis in children: clinical features, pathophysiology, and treatment. Immunol Allergy Clin North Am.

[166] He A, Feldman SR, Fleischer AB (2018). An assessment of the use of antihistamines in the management of atopic dermatitis. J Am Acad Dermatol.

[167] Nowicki RJ, Trzeciak M, Kaczmarski M, Wilkowska A, Czarnecka-Operacz M, Kowalewski C, Atopic dermatitis (2020). Interdisciplinary diagnostic and therapeutic recommendations of the polish dermatological society, polish society of allergology, polish pediatric society and polish society of family medicine. Part II. Systemic treatment and new therapeutic methods. Postepy Dermatol Alergol.

[168] Wollenberg A, Oranje A, Deleuran M, Simon D, Szalai Z, Kunz B (2016). ETFAD/EADV Eczema task force 2015 position paper on diagnosis and treatment of atopic dermatitis in adult and paediatric patients. J Eur Acad Dermatol Venereol.

[169] van Zuuren EJ, Apfelbacher CJ, Fedorowicz Z, Jupiter A, Matterne U, Weisshaar E (2014). No high level evidence to support the use of oral H1 antihistamines as monotherapy for eczema: a summary of a Cochrane Systematic review. System Rev.

[170] (http://www.agenziafarmaco.gov.it/sites/default/files/ IT_Hydroxyzine_start_of_referral.pdf)

[171] Matterne U, Böhmer MM, Weisshaar E, Jupiter A, Carter B, Apfelbacher CJ (2019). Oral H1 antihistamines as ‘add-on’ therapy to topical treatment for eczema. Cochrane Database Syst Rev.

[172] Kay GG (2000). The effects of antihistamines on cognition and performance. J Allergy Clin Immunol.

[173] Yu SH, Attarian H, Zee P, Silverberg JI (2016). Burden of sleep and fatigue in US adults with atopic dermatitis. Dermatitis.

[174] Schad CA, Skoner DP (2008). Antihistamines in the pediatric population: achieving optimal outcomes when treating seasonal allergic rhinitis and chronic urticaria. Allergy Asthma Proc.

[175] Food and Drug Administration HaHS (2002). Labeling of diphenhydramine-containing drug products for over-the-counter human use. Final rule. Fed Regist.

[176] Eichenfield LF, Tom WL, Berger TG, Krol A, Paller AS, Schwarzenberger K, Guidelines of care for the management of atopic dermatitis: section 2 (2014). Management and treatment of atopic dermatitis with topical therapies. J Am Acad Dermatol.

[177] Watanabe S, Narisawa Y, Arase S, Okamatsu H, Ikenaga T, Tajiri Y (2003). Differences in fecal microflora between patients with atopic dermatitis and healthy control subjects. J Allergy Clin Immunol.

[178] Penders J, Thijs C, van den Brandt PA, Kummeling I, Snijders B, Stelma F (2007). Gut microbiota composition and development of atopic manifestations in infancy: the KOALA Birth Cohort Study. Gut.

[179] Rook GA, Brunet LR (2005). Microbes, immunoregulation, and the gut. Gut.

[180] Kim J, Kim BE, Leung DYM (2019). Pathophysiology of atopic dermatitis: clinical implications. Allergy Asthma Proc.

[181] Kwon HK, Lee CG, So JS, Chae CS, Hwang JS, Sahoo A (2010). Generation of regulatory dendritic cells and CD4+Foxp3+ T cells by probiotics administration suppresses immune disorders. Proc Natl Acad Sci U S A.

[182] Akelma AZ, Biten AA (2015). Probiotics and infantile atopic eczema. Pediatric Health Med Ther.

[183] Makrgeorgou A, Leonardi‐Bee J, Bath‐Hextall FJ, Murrell DF, Tang MLK, Roberts A et al. Cochrane Skin Group. Cochrane Database Syst Rev. 2018;11(11):CD006135. 10.1002/14651858.CD006135.pub3 PMC6517242 30480774

[184] Kim JY, Park BK, Park HJ, Park YH, Kim BO, Pyo S (2013). Atopic dermatitis-mitigating effects of new Lactobacillus strain, Lactobacillus sakei probio 65 isolated from Kimchi. J Appl Microbiol.

[185] Park SB, Im M, Lee Y, Lee JH, Lim J, Park YH, Seo YJ (2014). Effect of emollients containing vegetable-derived lactobacillusin the treatment of atopic dermatitis symptoms: split-body clinical trial. Ann. Dermatol.

[186] Baldwin H, Aguh C, Andriessen A, Benjamin L, Ferberg AS, Hooper D (2020). Atopic dermatitis and the role of skin micobiome in choosing prevention, treatment and maintenance options. J Drugs Dermatol.

[187] Seitè S, Zelenkova H, Martin R (2017). Clinical efficacy of emollient in atopic dermatitis patients-relationship with the skin microbiota modification. Clin Cosmet Invest Dermatol.

[188] Gueniche A, Knaudt B, Schuck E, Volz T, Bastien P, Martin R (2008). Effect of nonpathogenic gram negative bacterium vitreoscilla filiformis lysate on atopic dermatitis: a prospective, randomized, double-blind, placebo-controlled clinical study. Br J Dermatol.

[189] Butler É, Lundqvist C, Axelsson J (2020). Lactobacillus reuteri DSM 17938 as a novel topical cosmetic ingredient: a proof of concept clinical study in adults with atopic dermatitis. Microorganisms.

[190] Holme SA, Anstey AV (2004). Phototherapy and PUVA photochemotherapy in children. Photodermatol Photoimmunol Photomed.

[191] Meduri NB, Vandergriff T, Rasmussen H, Jacobe H (2007). Phototherapy in the management of atopic dermatitis: a systematic review. Photodermatol Photoimmunol Photomed.

[192] Ortiz-Salvador JM, Pérez-Ferriols A (2017). Phototherapy in Atopic Dermatitis. Adv Exp Med Biol.

[193] Rodenbeck DL, Silverberg JI, Silverberg NB (2016). Phototherapy for atopic dermatitis. Clin Dermatol.

[194] Darrigade AS (2019). Traitements topiques et photothérapie dans la dermatite atopique: topical treatments and phototherapy in atopic dermatitis. Ann Dermatol Venereol.

[195] Farahnik B, Patel V, Beroukhim K, Zhu TH, Abrouk M, Nakamura M (2016). Combining biologic and phototherapy treatments for psoriasis: safety, efficacy, and patient acceptability. Psoriasis (Auckl).

[196] Cameron H, Yule S, Dawe RS, Ibbotson SH, Moseley H, Ferguson J (2014). Review of an established UK home phototherapy service 1998–2011: improving access to a cost-effective treatment for chronic skin disease. Public Health.

[197] Koek MB, Buskens E, Steegmans PH, van Weelden H, Bruijnzeel-Koomen CA, Sigurdsson V (2006). UVB phototherapy in an outpatient setting or at home: a pragmatic randomised single-blind trial designed to settle the discussion. The PLUTO study. BMC Med Res Methodol.

[198] Nakajima S, Nomura T, Common J, Kabashima K (2019). Insights into atopic dermatitis gained from genetically defined mouse models. J Allergy Clin Immunol.

[199] Harb H, Chatila TA (2020). Mechanisms of dupilumab. Clin Exp Allergy.

[200] Rohner MH, Thormann K, Cazzaniga S. Dupilumab reduces inflammation and restores the skin barrier in patients with atopic dermatitis. Allergy. 2020. Online ahead of print. 10.1111/all.14664 10.1111/all.14664 33210741

[201] Thomson J, Wernham AGH, Williams HC (2018). Long-term management of moderate-to-severe atopic dermatitis with dupilumab and concomitant topical corticosteroids (LIBERTY AD CHRONOS): a critical appraisal. Br J Dermatol.

[202] Thaçi D, Simpson E L, Deleuran M, Kataoka Y, Chen Z, Gadkari A (2019). Efficacy and safety of dupilumab monotherapy in adults with moderate-to-severe atopic dermatitis: a pooled analysis of two phase 3 randomized trials (LIBERTY AD SOLO 1 and LIBERTY AD SOLO 2). J Dermatol Sci.

[203] Simpson E, Paller AS, Siegfried EC (2020). Efficacy and safety of dupilumab in adolescents with uncontrolled moderate to severe atopic dermatitisa phase 3 randomized clinical trial. JAMA Dermatol.

[204] Calzavara-Pinton P, Belloni Fortina A, Bonamonte D, Marseglia GL, Miraglia Del Giudice M, Musarra A, Diagnosis and management of moderate to severe atopic dermatitis in adolescents (2021). A Consensus by the Italian Society of Dermatology and Venereology (SIDeMaST), the Italian Association of Hospital Dermatologists and Public Health (ADOI), the Italian Association of Hospital and Territorial Allergists and Immunologists (AAIITO), the Italian Society of Allergy, Asthma and Clinical Immunology (SIAAIC), the Italian Society of Pediatric Allergy and Immunology (SIAIP), the Italian Society of Allergological, Occupational and Environmental Dermatology (SIDAPA), and the Italian Society of Pediatric Dermatology (SIDerP). Ital J Dermatol Venerol.

[205] Paller AS, Siegfried EC, Thaçi D, Wollenberg A, Cork MJ, Arkwright PD (2020). Efficacy and safety of dupilumab with concomitant topical corticosteroids in children 6 to 11 years old with severe atopic dermatitis: a randomized, double-blinded, placebo-controlled phase 3 trial. J Am Acad Dermatol.

[206] Agache I, Akdis CA, Akdis M, Brockow K, Chivato T, Del Giacco S (2021). EAACI Biologicals Guidelines—dupilumab for children and adults with moderate-to-severe atopic dermatitis. Allergy.

[207] Katoh N (2021). Emerging treatments for atopic dermatitis. J Dermatol.

[208] Silverberg JI, Pinter A, Pulka G (2020). Phase 2B randomized study of nemolizumab in adults with moderate-to-severe atopic dermatitis and severe pruritus. J Allergy Clin Immunol.

[209] Puar N, Chovatiya R, Paller AS (2020). New treatments in atopic dermatitis. Ann Allergy Asthma Immunol.

[210] Guttman-Yassky E, Blauvelt A, Eichenfield LF, Paller AS, Armstrong AW, Drew J (2020). Efficacy and safety of lebrikizumab, a high-affinity interleukin 13 inhibitor, in adults with moderate to severe atopic dermatitis: a Phase 2b randomized clinical trial. JAMA Dermatol.

[211] Simpson E, Blauvelt A, Guttman-Yassky E, et al. Efficacy and safety of tralokinumab monotherapy in adult patients with moderate-to-severe atopic dermatitis: results from two 52-week Phase 3 trials (ECZTRA 1 and ECZTRA 2). Presented at the American Academy of Dermatology Virtual Meeting Experience (AAD VMX). 2020;4(6).

[212] Simpson EL, Parnes JR, She D, Crouch S, Rees W, Mo M (2019). Tezepelumab, an anti-thymic stromal lymphopoietin monoclonal antibody, in the treatment of moderate to severe atopic dermatitis: a randomized Phase 2a clinical trial. J Am Acad Dermatol.

[213] Guttman-Yassky E, Pavel AB, Zhou L, Estrada YD, Zhang N, Xu H (2019). GBR 830, an anti-OX40, improves skin gene signatures and clinical scores in patients with atopic dermatitis. J Allergy Clin Immunol.

[214] Guttman-Yassky E, Brunner PM, Neumann AU, Khattri S, Pavel AB, Malik K (2018). Efficacy and safety of fezakinumab (an IL-22 monoclonal antibody) in adults with moderate-to-severe atopic dermatitis inadequately controlled by conventional treatments: A randomized, double blind, Phase 2a trial. J Am Acad Dermatol.

[215] Fridman JS, Scherle PA, Collins R, Burn TC, Li Y, Li J (2010). Selective inhibition of JAK1 and JAK2 is efficacious in rodent models of arthritis: preclinical characterization of INCB028050. J Immunol.

[216] Simpson EL, Lacour JP, Spelman L, Galimberti R, Eichenfield LF, Bissonnette R (2020). Baricitinib in patients with moderate-to-severe atopic dermatitis and inadequate response to topical corticosteroids: results from two randomized monotherapy phase III trials. Br J Dermatol.

[217] Biggioggero M, Becciolini A, Crotti C, Agape E, Favalli EG (2019). Upadacitinib and filgotinib: the role of JAK1 selective inhibition in the treatment of rheumatoid arthritis. Drugs Context.

[218] Guttman-Yassky E, Thaci D, Pangan AL, Hong HC, Papp KA, Reich K (2020). Upadacitinib in adults with moderate to severe atopic dermatitis: 16-week results from a randomized, placebo-controlled trial. J Allergy Clin Immunol.

[219] Napolitano M, Fabbrocini G, Cinelli E, Stingeni L, Patruno C (2020). Profile of baricitinib and its potential in the treatment of moderate to severe atopic dermatitis: a short review on the emerging clinical evidence. J Asthma Allergy.

[220] Napolitano M, Ruggiero A, Fontanella G, Fabbrocini G, Patruno C (2020). New emergent therapies for atopic dermatitis: a review of safety profile with respect to female fertility, pregnancy, and breastfeeding. Dermatol Ther.

[221] https://www.accessdata.fda.gov/drugsatfda_docs/label/2019/211675s000lbl.pdf)

[222] Gooderham MJ, Forman SB, Bissonnette R, Beebe JS, Zhang W, Banfield C (2019). Efficacy and safety of oral janus kinase 1 inhibitor abrocitinib for patients with atopic dermatitis. A phase 2 randomized clinical trial. JAMA Dermatol.

[223] Rizk P, Rodenas M, De Benedetto A (2019). Allergen immunotherapy and atopic dermatitis: the good, the bad, and the unknown. Curr Allergy Asthma Rep.

[224] Pajno GB, Bernardini R, Peroni D, Arasi S, Martelli A, Landi M (2017). Clinical practice recommendations for allergen specific immnotherapy in children. The Italian Consensus report. Ital J Pediatrics.

[225] Muraro A, Roberts G, Halken S, Agache I, Angier E, Fernandez-Rivas M (2018). EAACI guidelines on allergen immunotherapy: Executive statement. Allergy.

[226] Ridolo E, Martignago I, Riario-Sforza GG, Incorvaia C (2018). Allergen immunotherapy in atopic dermatitis. Expert Rev Clin Immunol.

[227] Tam HH, Calderon MA, Manikam L, Nankervis H, Núñez IG, Williams HC (2016). Specific allergen immunotherapy for the treatment of atopic eczema: a cochrane systematic review. Allergy.

[228] Liu L, Chen J, Xu J, Yang Q, Gu C, Ni C (2019). Sublingual immunotherapy of atopic dermatitis in mite –sensitized patients: a multi-centre, randomized, double blind, placebo controlled study. Artif Cells Nanomed Biotechnol.

[229] Galli E, Chini L, Nardi S, Benincori N, Panei P, Fraioli G (1994). Use of oral hyposensitization therapy to Dermatophagoides pteronyssinus in children with atopic dermatitis. Allergolol Immnophatol (Madr).

[230] Pajno GB, Caminiti L, Vita D, Barberio G, Salzano G, Lombardo F (2007). Sublingual immunotherapy in mite-sensitized children with atopic dermatitis: a randomized, double-blind, placebo-controlled study. J Allergy Clin Immunol.

[231] Eichenfield LF, Ahluwalia J, Waldman A, Borok J, Udkoff J, Boguniewicz M (2017). Current guidelines for the evaluation and management of atopic dermatitis: a comparison of the joint task force practice parameter and american academy of dermatology guidelines. J Allergy Clin Immunol..

[232] Chu H, Park KH, Kim SM, Lee JH, Park JW, Lee KH (2020). Allergen-specific immunotherapy for patients with atopic dermatitis sensitized to animal dander. Immun Inflamm Dis.

[233] Cacciapuoti S, Luciano MA, Megna M, Annunziata MC, Napolitano M, Patruno C (2020). The role of thermal water in chronic skin diseases management: a review of the literature. J Clin Med.

[234] Harari M (2012). Climatotherapy of skin diseases at the dead sea – an update. Anales de Hidrología Médica.

[235] Heinlin J, Schiffner-Rohe J, Schiffner R, Einsele-Krämer B, Landthaler M, Klein A (2011). A first prospective randomized controlled trial on the efficacy and safety of synchronous balneophototherapy vs narrow-band UVB monotherapy for atopic dermatitis. J Eur Acad Dermatol Venereol.

[236] Merial-Kieny C, Castex-Rizzi N, Selas B, Mery S, Guerrero D (2011). Avène thermal spring water: an active component with specific properties. J Eur Acad Dermatol Venereol.

[237] Pigatto P (2005). The efficacy of avène thermal spring water in light to moderate atopicdermatitis. Ann Dermatol Venereol.

[238] Giannetti A (2005). The hydrotherapy centre in avène-les-bains a controlled study in atopic dermatitis. Ann Dermatol Venereol.

[239] Taieb C, Myon E (2005). Dermatite atopique: Impact de l’hydrothèrapie sur la qualite’ de vie. Ann Dermatol Venereol.

[240] Dikova A (2016). An observational study on patients suffering from atopic dermatitis undergoing balneotherapy. Proceeding in J Am Acad Dermatol.

[241] Farina S, Gisondi P, Zanoni M, Pace M, Rizzoli L, Baldo E (2011). Balneotherapy for atopic dermatitis in children at Comano spa in Trentino, Italy. J Dermatolog Treat.

[242] Guerrero D, Garrigue E (2017). Eau thermale d’Avène et dermatite atopique: avène’s thermal water and atopic dermatitis. Ann Dermatol Venereol.

[243] Martin H, Laborel-Préneron E, Fraysse F, Nguyen T, Schmitt AM, Redoulès D (2016). Aquaphilus dolomiae extract counteracts the effects of cutaneous S. aureus secretome isolated from atopic children on CD4 T cell activation. Pharm Biol.

[244] Aries MF, Hernandez-Pigeon H, Vaissière C, Delga H, Caruana A, Lévêque M (2016). Anti-inflammatory and immunomodulatory effects of Aquaphilus dolomiae extract on in vitro models. Clin Cosm Investig Dermatol.

[245] Seité S (2013). Thermal waters as cosmeceuticals: La Roche Posay thermal spring waterexample. Clin Cosmet Investing Dermatol.

[246] Zeichner J, Seite S (2018). From probiotic to prebiotic using thermal spring water. J Drugs Dermatol.

[247] Pedron R, Esposito A, Bianconi I, Pasolli E, Tett A, Asnicar F (2019). Genomic and metagenomic insights into the microbial community of a thermal spring. Microbiome.

[248] Galli E, Neri I, Ricci G, Baldo E, Barone M, Belloni Fortina A (2016). Consensus conference on clinical management on pediatric atopic dermatitis. Ital J Pediatr.

[249] Piano nazionale della cronicità. A cura di Direzione generale della Programmazione sanitaria - Ministero della Salute. Anno. 2016.

[250] Olsson M, Bajpai R, Yew YW, Koh MJA, Thng S, Car J (2020). Association between health-related quality of life and health-related costs among children with atopic dermatitis and their caregivers: across-sectional study. Pediatr Dermatol.

[251] Maksimovic N, Zaric M, Reljic V, Nikolic M, Gazibara T (2020). Factors associated with improvement of quality of life among parents of children with atopic dermatitis:1-year prospective cohort study. Eur Acad Dermatol Venereol.

[252] Bass AM, Anderson KL, Feldman SR (2015). Interventions to increase treatment adherence in pediatric atopic dermatitis: a systematic review. J Clin Med.

[253] Peters EM, Michenko A, Kupfer J, Kummer W, Wiegand S, Niemeier V (2014). Mental stress in atopic dermatitis– neuronal plasticity and the cholinergic system are affected in atopic dermatitis and in response to acute experimental mental stress in a randomized controlled pilot study. PLoS One.

[254] Dalgard FJ, Gieler U, Tomas-Aragones L, Lien L, Poot F, Jemec GBE (2015). The psychosocial burden of skin diseases: a cross-sectional multicenter study among dermatological out-patients in 13 European countries. J Invest Dermatol.

[255] Ersser SJ, Cowdell F, Latter S, Gardiner E, Flohr C, Thompson AR (2014). Psychological and educational interventions for atopic eczema in children. Cochrane Database Syst Rev.

[256] Eicher L, Knop M, Aszodi N, Senner S, French LE, Wollenberg A (2019). A systematic review of factors influencing treatment adherence in chronic inflammatory skin disease - strategies for optimizing treatment outcome. J Eur Acad Dermatol Venereol.

[257] El Hachem M, Di Mauro G, Rotunno R, Giancristoforo S, De Ranieri C, Carlevaris CM (2020). Pruritus in pediatric patients with atopic dermatitis: a multidisciplinary approach - summary document from an Italian expert group. Ital J Pediatr.

[258] Leibovic V, Magora F, Cohen S, Ingber A (2009). Effects of virtual reality immersion and audiovisual distraction techniques for patients with pruritus. Pain Res Manag.

